# Developing and Testing the Feasibility of a Culturally Based Tele-Palliative Care Consult Based on the Cultural Values and Preferences of Southern, Rural African American and White Community Members: A Program by and for the Community

**DOI:** 10.1089/heq.2019.0120

**Published:** 2020-03-26

**Authors:** Ronit Elk, Linda Emanuel, Joshua Hauser, Marie Bakitas, Sue Levkoff

**Affiliations:** ^1^ Department of Medicine, School of Medicine, University of Alabama at Birmingham, Birmingham, Alabama.; ^2^ Department of Medicine, Feinberg School of Medicine, Northwestern University, Evanston, Illinois.; ^3^ Department of Acute, Chronic and Continuing Care, School of Nursing, University of Alabama at Birmingham, Birmingham, Alabama.; ^4^ College of Social Work, University of South Carolina, Columbia, South Carolina.

**Keywords:** culture, palliative care, CBPR (community-based participatory research), rural, African American, telehealth

## Abstract

**Purpose:** Lack of appreciation of cultural differences may compromise care for seriously ill minority patients, yet culturally appropriate models of palliative care (PC) are not currently available in the United States. Rural patients with life-limiting illness are at high risk of not receiving PC. Developing a PC model that considers the cultural preferences of rural African Americans (AAs) and White (W) citizens is crucial. The goal of this study was to develop and determine the feasibility of implementing a culturally based PC tele-consult program for rural Southern AA and W elders with serious illness and their families, and assess its acceptability to patients, their family members, and clinicians.

**Methods:** This was a three-phase study conducted in rural Beaufort, South Carolina, from January 2013 to February 2016. We used Community-Based Participatory Research methods, including a Community Advisory Group (CAG) with equal numbers of AA and W members, to guide the study. Phase 1: Cultural values and preferences were determined through ethnic-based focus groups comprising family members (15 W and 16 AA) who had cared for a loved one who died within the past year. We conducted a thematic analysis of focus group transcripts, focused on cultural values and preferences, which was used as the basis for the study protocol. Phase 2: Protocol Development: We created a protocol team of eight CAG members, two researchers, two hospital staff members, and a PC physician. The PC physician explained the standard clinical guidelines for conducting PC consults, and CAG members proposed culturally appropriate programmatic recommendations for their ethnic group for each theme. All recommendations were incorporated into an ethnic-group specific protocol. Phase 3: The culturally based PC protocol was implemented by the PC physician via telehealth in the local hospital. We enrolled patients age ≥65 with a life-limiting illness who had a family caregiver referred by a hospitalist to receive the PC consult. To assess feasibility of program delivery, including its acceptability to patients, caregivers, and hospital staff, using Donebedian's Structure-Process-Outcome model, we measured patient/caregiver satisfaction with the culturally based consult by using an adaptation of FAMCARE-2.

**Results:** Phase 1: Themes between W and AA were (1) equivalent: for example, disrespectful treatment of patients and family by hospital physicians; (2) similar but with variation: for example, although religion and church were important to both groups, and pastors in both ethnic groups helped family face the reality of end of life, AA considered the church unreservedly central to every aspect of life; (3) divergent, for example, AAs strongly believed that hope and miracles were always a possibility and that God was the decider, a theme not present in the W group. Phase 2: We incorporated ethnic group-specific recommendations for the culturally based PC consult into the standard PC consult. Phase 3: We tested feasibility and acceptability of the ethnically specific PC consult on 18 of 32 eligible patients. The telehealth system worked well. PC MD implementation fidelity was 98%. Most patients were non-verbal and could not rate satisfaction with consult; however, caregivers were satisfied or very satisfied. Hospital leadership supported program implementation, but hospitalists only referred 18 out of 28 eligible patients.

**Conclusions:** The first culturally based PC consult program in the United States was developed in partnership with AA and W Southern rural community members. This program was feasible to implement in a small rural hospital but low referral by hospitalists was the major obstacle. Program effectiveness is currently being tested in a randomized clinical trial in three southern, rural states in partnership with hospitalists. This method can serve as a model that can be replicated and adapted to other settings and with other ethnic groups.

## Introduction

Culture fundamentally shapes how individuals make meaning out of illness, suffering, and dying,^1^ and it strongly influences people's responses to diagnosis, illness, and treatment preferences.^1–3^ Considering patients' and families' culture is essential in all aspects of palliative care (PC). A lack of sensitivity to cultural differences may compromise end-of-life care for minority patients.^4^ However, culturally appropriate models of care that consider the diverse cultural preferences of seriously ill rural patients and their family caregivers are not currently available in the United States. There is an urgent need for research that emphasizes varying end-of-life care cultural preferences.^4–10^

The triple threat of rural geography, racial inequities, and older age hinders access to high-quality PC for rural Americans. In a state-by-state report card,^11,12^ the Southeastern United States, where a there is a significant proportion of rural dwellers and African Americans (AAs), PC access was ranked the lowest in the nation. Rural patients with life-limiting illness are at high risk of not receiving appropriate care due to a lack of health professionals (nearly two-thirds of rural U. S. counties are designated health professional shortage areas),^13^ long distances to treatment centers,^14^ and limited PC clinical expertise.^15^ Seventy-five percent of South Carolina (SC) is rural, and 34% of its residents reside in rural areas,^16^ where poverty and unemployment rates are high and per capita income is low.^17^ AAs comprise 36% of those who live in rural SC.^18^

The lack of PC services in rural settings is evident in the lack of guidance by national organizations to address the unique challenges and barriers faced by rural patients.^19^ The clinical guidelines on quality PC from the National Consensus Project^20^ did not contain the term “rural” through the first three editions, nor did they address how these standards should be applied in rural settings.^21^ Geographic inequities in access to PC are expected to rise as the rural population ages and the demand for PC increases.^22^ Rural patients with life-limiting illness remain vulnerable and at high risk of not receiving appropriate care. During the past few months of life, rural patients may experience significant and unnecessary suffering that an accessible PC consult could have alleviated; this is a major disparity for seriously ill rural patients.^7^ The need for research to guide best practices in providing PC to rural patients is pressing.^21^

Even when palliative and hospice services are available, AAs, when compared with Whites, are more likely to receive medically ineffective, poor quality, and high-cost care, due to general mistrust of health care providers and a fragmented health care system that is generally insensitive to cultural differences that can guide treatment choices.^23–28^ Despite proven effectiveness, numerous studies have shown that AAs underutilize palliative and hospice care.^29–34^ Suggested reasons for this include both a lack of exposure to hospice or PC information^35–37^ and possibly differences in values for end-of-life care.

Historically, end-of-life care has been rooted in White middle class cultural and religious values,^4,36^ with its very different frame of reference, value system, and life experience compared with many AAs.^38^ Where middle class Whites may emphasize individual choice, AA values support family-centered decision making.^7^ Faith, spiritual beliefs, and guidance of a spiritual leader^36^ are very meaningful to AAs, especially as they cope with illness and make treatment decisions.^39,40^ However, physicians rarely ask patients about their spirituality.^41^ AAs rely on hope^42^ and faith in God's healing power:^43^ This can be at odds with physicians' felt need to share a terminal prognosis.^31^

Inpatient PC consultations have identified unrecognized symptoms and unmet needs,^44–48^ and they have been associated with fewer intensive care unit (ICU) days^49,50^ and ICU deaths,^51^ and improved care processes and higher rates of documentation of goals of care.^29,30,52^ However, lack of access to palliative consultations results in less availability of PC benefits to rural and minority patients.

Finally, and most significantly, historical and social factors, including slavery, racism, medical experimentation and exploitation,^31,53^ as well as ongoing racism and microaggressions,^54^ have left a deep-seated legacy of mistrust in the AA community.^55–57^ A recent report^58^ found that AAs and Whites were “worlds apart” in their perceptions of racial equality and actual gaps in household income. This is even more strongly felt in the “Deep South” where slavery was promoted.^59^ A recent study^60^ found that AAs are more likely than other racial groups to believe that physicians did not care about them as individuals, and were less likely to trust their physicians' judgment and personal competence.

The overall goal of this three-phase study was to develop and determine the feasibility of implementing a culturally based PC tele-consult program for rural Southern AAs and White elders with serious illness and their families, and assess its acceptability to patients, their family members, and clinicians. The aims by phase were: Phase 1: To determine cultural values and preferences of each ethnic group and ascertain ethnic group commonalities and differences. Phase 2: To develop a culturally based PC consult protocol across ethnic groups. Phase 3: To determine feasibility of program delivery, and acceptability to patients, caregivers, and clinicians, and patient/caregiver satisfaction with the culturally based consult.

## Methods

The study was conducted at Beaufort Memorial Hospital (BMH) in Beaufort, SC. Beaufort County has a population of 175,852, and most of Beaufort County's census tracts are rural.^61^ IRB approval for all three phases of the study was obtained from the University of South Carolina Institutional Review Board, and for Phase 3, from the Beaufort Memorial Hospital.

### Study design

**FIG. 1.**
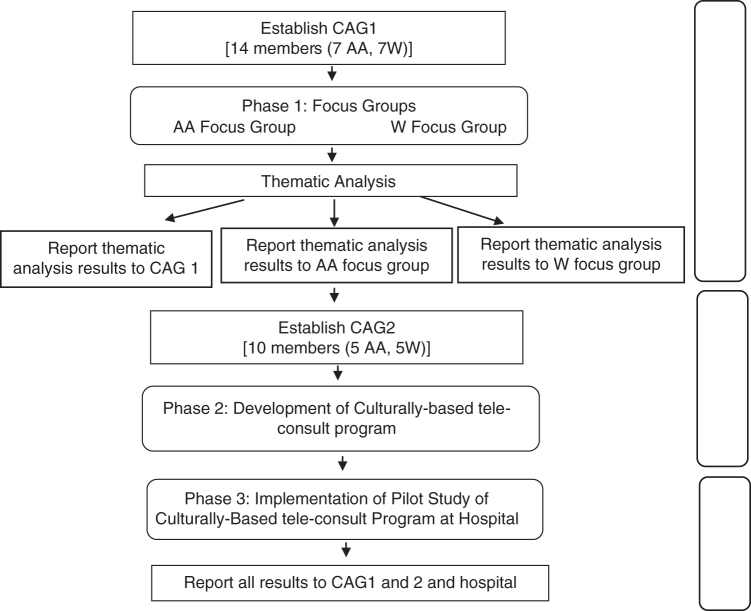
Study design.

This three-phase study used Community Based Participatory Research (CBPR) methods and principles, as illustrated in Figure 1, to define the study protocol and identify feasibility of a culturally based PC consultation to improve PC access. CBPR, a public health method in which academia and the community form a joint partnership to address community issues, has been found to address health disparities^62,63^ and result in demonstrable positive health outcomes.^64^

CBPR builds on community strengths; the community is integral to all phases of the research, for the mutual benefit of all partners involved in the process, and in disseminating findings and knowledge to all partners.^65^ Consumer input has been demonstrated to enhance both the quality and acceptability of interventions. CBPR has been recommended as a promising strategy for PC research that aligns with the priorities of stakeholders, as a means to deliver tailored and appropriate care to underserved communities.^66^

Phase 1 engaged community members in the planning phase, serving as the first step in forming relationships and building trust with the community, and in obtaining community guidance into the study. Community Advisory Group (CAG) members were recruited with the assistance of the community health educator at BMH who had strong ties with community groups.

The CAG included equal numbers of AA (*n*=7) and White (*n*=7) members. The AA members were a county council member, leader of the Gullah Church Nurses Association, a pastor of a local AA church, a hospital employee, a hospice social worker, and a community member whose wife had recently died. White members included the nursing director of the BMH Cancer Center, its community health educator, a local social worker, two local hospice staff, and a community member whose loved one had recently died.

### Phase 1: CAG planning and focus groups

The CAG planning meetings for Phase 1 (1.5 h each) were held over 2 months at BMH. The CAG members received an honorarium for their time ($25/meeting) and a gas card ($25/meeting) for travel.

#### Focus groups

In designing the research protocol, we chose focus groups as the most appropriate data gathering method for the study, as past research has suggested they provide an atmosphere that is the most conducive to respondents in sharing their personal experiences.^67–69^ We presented our rationale for this decision and elicited CAG feedback as to the appropriateness. CAG members were familiar with focus groups; this method had been widely used in Beaufort in an assessment of health care needs,^70^ and considered this method appropriate. The CAG members provided input into culturally appropriate ways to recruit for, plan, and run the focus groups. The AA members strongly recommended holding separate focus groups by ethnicity, making it clear that AAs would be more comfortable and more likely to speak openly if focus groups were limited to AAs. As a result, we revised the original consent forms to hold separate focus groups.

CAG members designed the recruitment flyer, using a design that was considered appropriate for each community, and distributed these to churches, hospitals, hospices, and other community settings. Local newspapers were sent information, and the principal investigator (PI) and CAG members were interviewed for three articles published. The CAG-developed screening protocol was used to determine eligibility of potential callers. The CAG members recommended that focus groups be held in a well-respected but neutral space. Meeting dates and times were weekdays after 5:00 pm, lasting no more than an hour and a half, and included a light dinner.

#### Focus group inclusion criteria

To be eligible to participate in the focus group, respondents had to be a family member of a loved one aged 60 and older who had died in the previous 12 months after an illness of 3 or more months, and in whose care they had been involved. Both the family member and loved one had to be Beaufort County residents, and the loved one had to have been treated in Beaufort County (with the possibility of occasional visits to larger hospitals in Charleston or Savannah). Thirty-one focus group members were recruited (15 White and 16 AA) (Table 1). Two thirds were immediate family; all provided at least 3 h of care/day to their loved one, with half providing 12 or more h of care per day.

**Table 1.** Phase 1: Demographics of Focus Group Participants

	White, *n* (%)	AA, *n* (%)	Total	Percent of sample
Participated in study	15	16	31	100
Years in Beaufort County				
<5	0 (0)	2 (12.5)	2	6.45
5–15	7 (46.6)	1 (6.25)	8	25.81
16–25	4 (26.6)	2 (12.5)	6	19.35
>25	4 (26.6)	11 (68.75)	15	48.39
Relationship to loved one^a^				
Immediate family	14 (93.3)	10 (62.5)	24	77.4
Friends and extended family	1 (6.67)	6 (37.5)	7	22.5
Hours cared for loved one per day				
3	3 (20)	3 (18.75)	6	19.35
4–8	3 (20)	3 (18.75)	6	19.35
8–12	1 (6.67)	1 (6.25)	2	6.45
12 or more	8 (53.33)	9 (56.25)	17	54.84
Location of passing				
Hospital	3 (20)	2 (12.5)	5	16.13
Home	8 (53.33)	12 (75)	20	64.52
Nursing home	3 (20)	0 (0)	3	9.68
Hospice facility	1 (6.67)	2 (12.5)	3	9.68

^a^ χ^2^(1, *N*=31)=4.21, *p*=0.040.

AA, African American.

#### Focus group guidelines

Focus groups were conducted separately by ethnic group. Facilitators were of the same ethnicity as the group they facilitated, consistent with focus group methodology and as has been used successfully elsewhere.^71^ Planning meetings to develop the guidelines for the focus groups were held with the PI, two co-investigators (Co-Is), and the two facilitators. Both facilitators (one faculty member and one doctoral student) had received training in conducting focus groups. The protocol included ground rules (e.g., confidentiality), an explanation of why focus groups were being held separately, and the questions (and their probes) posed to focus group participants over the two meetings.

Questions were designed to focus on topics that impact end-of-life care. In this article, we focus on family members' preferences toward the care their loved one received in the 12 months before their death. Questions on this topic included: (1) “What did you find to be particularly helpful about the care or treatment your loved one received during their end-of-life care?” (2) “What did you like or not like about the way the healthcare staff communicated with your loved one/the family?” (3) “During the course of your loved one's illness, whose advice did you seek or who helped in making decisions about your loved one?” and (4) “During the end-of-life care experience with your loved one, whom among the healthcare staff did you trust?”

#### Focus group meetings

Two focus groups, a week apart, were held for each ethnic group at the local University in a comfortable meeting room. A light dinner was provided. The community health educator greeted each participant and introduced the PI and focus group facilitator. Respondents were presented with two IRB-approved study consent forms: the first to participate in the focus group study, and the second to be contacted for follow-up and potential participation in the next phase of the study. The facilitator started the meeting by introducing herself, the note takers, and the social worker who remained outside the room during all focus group meetings in case a participant(s) required professional support, and the two note takers sat at the back of the room.

Notes and tape recorders were transferred for storage in a locked, coded box to a locked data-storage room at the University. All tapes were transferred to Verbal Ink, a transcription firm. All transcriptions were kept on secured computers accessible only to the PI and study coordinator. All questionnaires were kept in locked university storage, with a unique identifier assigned to each questionnaire.

#### Data analysis

Analysis of the data followed standard procedures for qualitative data analysis;^72,73^ that is, systematic thematic analysis of transcripts identifying major and minor thematic areas; coding categories using open, axial, and selective coding; and sorting the data into coded categories, construct, and name typologies to describe family members' preferences for end-of-life care.^67,68,73^ A theme was defined as an issue raised or discussed by at least two or more members in that focus group. Themes were identified for coding based on the repetition of specific words, phrases and opinions, use of language and general thought patterns, as well as specific topics that dominated the focus group discussion.

Coding was conducted separately for each focus group meeting by two independent raters (R.E. and S.L.). Each independently reviewed and coded the data for themes, and then identified agreement. This was repeated for each of the four focus group meetings. In cases where there was no agreement, a discussion was held between the two raters and a Co-I (S.L.) experienced in focus group analysis, to reach consensus. Each agreed-upon theme and sub-theme was clearly defined, and several illustrative quotes were provided. Each theme was examined to determine whether it was similar across the two groups or whether it varied by group, and if so, in what way.

#### Report to CAG

After completion of the thematic analysis, results of each focus group were presented back separately to each of the focus groups, and all results were reported to CAG.

### Phase 2: CAG planning and development of culturally based PC tele-consult

#### Expansion of CAG

To expand community input and to compensate for some CAG member withdrawal, the CAG was expanded to include six additional members, three from each of the focus groups (CAG2). Focus group members added were selected among participants who had agreed to be re-contacted, had participated fully in the focus group meetings, and who held varying perspectives and opinions on a range of topics.

CAG2 met monthly for 28 months with the research team, which included the study PI (R.E.), the PC physician (J.H.) who participated remotely via Go-To-Meeting, and the study coordinator. Meetings were held on a Thursday evening at BMH, and a light dinner was provided. The CAG2 members received the same reimbursement as in Phase 1.

**FIG. 2.**
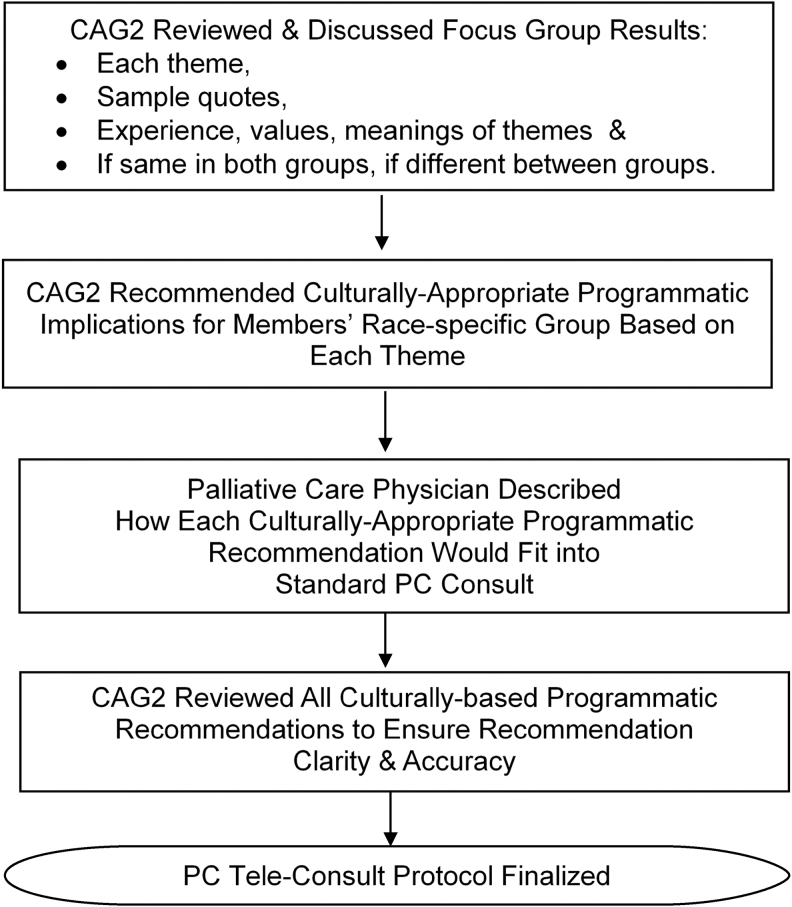
Phase 2 developing the culturally based PC tele-consult program. PC, palliative care.

Figure 2 illustrates the process used in developing the culturally based tele-consult. (1) First, the PC physician described the elements of a Standard Palliative Care Consult^20^ to provide an overview of where the community recommendations would fit. (2) The CAG2 reviewed each of the Phase 1 themes, including sample quotes that illustrated each them. Following an overview of all the themes, each theme was reviewed and discussed individually. (3) For each theme, and for their ethnic group, CAG2 members recommended culturally appropriate programmatic recommendations for the culturally based PC consult that they were designing. If the theme applied to both groups equally, all members made these recommendations, and if it was applicable to one or other group only, members of that group made the recommendations. (4) All the recommendations were reviewed to ensure an accurate portrayal of what CAG2 recommended, and those that did, were written into a study protocol for the study team and the PC physician to adhere to. The group discussed those that were not accurately portrayed further, until the representation was accurate.

### Phase 3: CAG planning and implementation of the pilot study culturally based PC tele-consult

Few, if any, research studies take place in such small rural hospitals and a hospital “gatekeeper,” the Chief Nursing Officer, opened several doors to approach the hospital CEO. Approval was obtained from the hospital CEO, and after a formal presentation to their IRB, approval for this study was given. Presentations were made at hospitalists' meetings by the PC physician and the study PI on at least two occasions to explain the study aims and methods. Cards with eligibility criteria were created and handed out to all hospitalists. The Study Coordinator was present at the hospital every day and built relationships with floor nurses, to whom she explained the study and gave out eligibility cards.

Eligible patients were only approached after permission from the hospitalists was obtained by means of an order for a PC consult from the hospitalist. Based on CAG recommendations, CAG members were the first to meet the patient and family members to explain the study to them. If there was interest in the study, CAG members introduced the study coordinator who completed the consent process.

All PC tele-consults were conducted in the patient's hospital room at a time convenient to the patient and family members. The telehealth computer was brought in by the study coordinator who remained in the room with the family during the consult. After the consult, the PC physician charted his findings and recommendations, which hospitalists could review. The study coordinator alerted the hospitalist that the report was in the chart, and it was the hospitalists' decision whether to implement PC physician's additional recommendations if any were made.

Midway through Phase 3, to enhance hospitalists' referral and involvement, we implemented a system whereby the PC physician texted the hospitalist by using a secure health care communication method, after the consult and offered to discuss the consult by phone if the hospitalist wished.

Feasibility of program delivery, including its acceptability to patients, caregivers, and hospital staff, was assessed by using Donebedian's Structure-Process-Outcome model.^74^ In this model, Structure examines the capacity of the care system (i.e., support of hospital staff and leadership, audiovisual equipment to access PC), Process evaluation addresses implementation fidelity to culturally based protocol, and Outcome evaluates acceptability of the program to patients and family and the effects of program on perceived patient and family satisfaction with the PC Consult (primary outcomes). Variables derived from this framework were measured through process evaluations (structure and process) and adaptation of FAMCARE-2 questionnaire (outcomes).

## Results

### Phase 1: focus groups

#### Calls to study hotline to participate in focus groups

Eighty-nine calls were received. Referral sources varied significantly between the two ethnic groups; the majority of Whites read about the study through articles in one of two local newspapers. Less than half of AAs called as a result of this source; most were referred directly by CAG members or through flyers placed in local churches by the CAG.

#### Exclusion

Eleven White callers and one AA did not meet eligibility criteria for focus group participation (primarily because their loved one had been treated in other parts of the United States).

#### Recruitment to focus groups

Due to higher than expected interest, we expanded our focus group numbers beyond our initially planned for 10 per group, and closed participation after consent was obtained from 15 Whites and 17 AAs.

#### Attendance at focus groups

All Whites (*n*=15) and all but one AA (*n*=16) who met eligibility criteria attended focus groups. There was considerable participation in both groups from all participants. Although a range of powerful emotions were shared, no one required the services of the social worker. Identifying data are presented in Table 1.

Relationships to loved ones varied significantly between the two groups. Most loved ones who had passed away in both groups were either parents or spouses, but there were more non-immediate family and friends in the AA group (i.e., great uncle, second cousin, godfather, and two close friends), supporting prior studies that document extended family members and fictive kin (unrelated by birth or marriage but with a significant relationship with the person, that are like a family member), as important members of AA families.^75–77^

#### Thematic analysis of focus group data

Ten themes, each with one or more sub-themes, emerged from the focus group data, and fell into two broad categories: (1) Experiences that patients and family members had at various treatment centers (physician offices, hospitals, hospices, nursing homes) during the care of their loved ones; for example, physician communication, pain medication; and (2) Cultural values, beliefs, and preferences; for example, the role of religion and church, family caregiving, cultural aspects of discussing death, and discussing prognosis.

Comparison of Themes Between Ethnic Groups: In eight of the themes, there were differences between the two ethnic groups within the sub-themes; one theme arose only among AAs (lack of trust) and one theme among Whites only (physician lack of respect for patients/family). All themes are described next.

### Phase 1 themes and Phase 2 recommendations

As the recommendations made by the CAG in Phase 2 are built/depend directly on the themes in Phase 1, we present the results of both phases by the themes given next (Tables 2–11).

**Table 2.** Theme: Lack of Trust in the Health Care System

Sub-theme(s)	Phase 1: focus groups		Phase 2: recommendations	
	AA	White	AA	White
(A) Distrust of medical system and physicians	“… that death hospital. That's what I call the hospital in O [small town in South Carolina] the death hospital. If you wanna’ die, go there. You will die.” I just wish I would have known honestly, what my grandfather died of, just put it on the paper …. Was it some sort of medication that you guys gave him that called it, you know, the end?”	X	Role of CAG (1) AA CAG member will be the first to meet and greet the AA hospitalized patients eligible to participate in the culturally based tele-consult and their families. (2) CAG members will explain how the study program was developed by the community for their community by incorporating culturally appropriate values and preferences. (3) After a short discussion and answering questions, CAG members will introduce the patient and family to the Study Coordinator. Role of Physician (1) Never call the patient and/or family member by first name, unless invited to do so. (2) Build in additional time to get to know the patient and family. Learn something about the patient and the family and during the consult conversation, discuss it so that the family knows you heard it and it was not just a rote gesture. (3) During the conversation, talk about something local to indicate familiarity with region where the patient resides (an indication that the physician knows something about the area).	Role of CAG (1) Although this theme did not arise in the White group, the recommendations were the same for the CAG, that is, CAG members will be the first to greet the eligible hospitalized patients and their families, although the ethnicity of the CAG member meeting with the patient and family was not specified. (2) Physician: Establish relationship with patient and family as a first step.

CAG, Community Advisory Group.

**Table 3.** Theme: Discomfort with Telehealth

Sub-theme(s)	Phase 1: focus groups		Phase 2: recommendations	
	AA	White	AA	White
(A) Uncomfortable with telehealth	“I'm just speaking for me, but some of the older ones wouldn't like it because a lot of people don't use computers. Some people do and some people don, you know, so it would be sort of difficult for some of our older people who don't … who is not computer literate at the time ….” “And like you said with older people, especially African American, the computer is not there yet. And as far as Skype goes, I don't even like to Skype too much unless my hair's right.”	“I'm a person who wants everything simple, right in front of me. That's [telehealth] is very foreign to me. Very foreign.” “… I guarantee that my husband would not have been comfortable with it. My husband was 15 years older than I am, and I do think that it is something of an age [issue].”	(1) Wear white coat. (2) Start session by acknowledging that this method (telehealth) is not the same as sitting opposite, or next to, one another.	(1) Wear white coat. (2) Start session by acknowledging that this method (telehealth) is not the same as sitting opposite, or next to, one another.
(B) It could work with these provisions OR under these conditions	“If it's an agency or someone you have a relationship with already, where there's an established record of you, but you may come into crisis which we always do during these final days, and if that's the case, then you can have someone to listen.” “I think the telecommunication … has its advantage to some degree because we have transplanted families everywhere … but it's also good for that person to have the opportunity to talk with whoever it is taking care of that family member.”	“You know, I think it could be helpful … I think that the first meeting, two, three, should be certainly in person, should be with a medical professional that you trust, and that you have a good relationship with … But then after that I think to not have to take J. out of the house, that to me would be fabulous.” “… we talked last week about how impersonal some of the people felt their doctors were, and that's what I think people are reaching out for. They want somebody that, whether they do or not … acts like they care, so I think that would be a really big plus to do it but have your physician there with you.”	(1) Continue relationship with current doctor (hospitalist). (2) Have someone from the study in the room to ensure continuity. (3) Family member should be in room with the patient.	(1) Continue relationship with current doctor (hospitalist). (2) Have someone from the study in the room to ensure continuity. (3) Family member should be in room with the patient.

**Table 4.** Theme: Treat Family and Patient with Respect

Sub-theme(s)	Phase 1: focus groups		Phase 2: recommendations	
	AA	White	AA	White
(A) Experiences of physicians acting in an insensitive or rude manner	X	“It wasn't our regular doctor, this man comes out in the waiting room and there's our church members and … N's mother and his sister and daughters and everybody out there. The waiting room was full. He says, ‘Well,’ he said, “I don't know how he's lived this long and it'll be a miracle if he's here at Thanksgiving,” and this was the end of September. “His heart and lungs are just shot and it's just a miracle that he's still living.” “I was trying to get the doctor to understand our insurance, and he says, ‘You know, usually when I give a patient a prescription and they go get it and they pay their $2,000, but here I have one who comes in and wants it for nothing.’ So I went to the pharmacy, called the insurance agency, told them what I was up against and he fixed it all. I got the T [medication.] I went back and he saw the bag in my hand, the doctor, and he says, ‘well, these insurance companies give you a hard time, don't they?’ I said, ‘it wasn't the insurance company. You were rude! What gives them a right to behave like that when you're under so much stress anyway?’ ”	(1) Although this theme did not arise in the AA group, the recommendations were that physicians should be courteous, and respectful of confidentiality.	(1) Physicians should never be rude, always be courteous.

**Table 5.** Theme: Religion, Church, and Pastor

Sub-themes	Phase 1: focus groups		Phase 2: recommendations	
	AA	White	AA	White
(A) Religion is our source of comfort and the church is at the center of our lives	“My son in law is a minister, so he called … and I asked him to please pray for him [patient] over the phone. So I put the phone to his ear, and I put the speaker on so we could both hear, and he prayed, and when he finished, my husband said ‘Amen’ right along with him and that was the last word he ever said. After that prayer he never said another word.” “And so because you encounter things and people will question you as to how you go through it or how you handled it; because it really has nothing to do with you; it has to do with your faith … because it is rough. You know, because it's someone you love and even though we know what the Word says about it, you really hate to part with that person.” “She [sister] said, ‘I mean you're tired’ and she said, ‘You just go ahead with the Lord.’ And my dad opened his eyes, and my sisters and I were standing around the bed, and he opened his eyes and he turned around and looked at all of us. He took that last breath and just went right on to sleep.”	X	(1) Religion is the source of all of comfort, a key value, and it's the perspective from which AAs view the world. Therefore, in all PC physician interactions with AA patients, recognize and respect that this is an integral part of all that is said and done.	X
(B) Pastor helped accept end-of-life reality	“And at Sabbath, they was in service at church and my pastor was there … and I just started screaming that afternoon. I mean just boo-hoo, crying like someone was killing me, and he came over to me and he prayed. And he said, ‘God said to tell you that you've done all that you can do. So you've done everything you could do for him.’ ” “Those last two weeks, I mean he just went down, and when I had—my daughter and son-in-law had gone to a football game and so—my son-in-law is a minister, so he called after they got back from the game and I asked him to please pray for him over the phone. So I put the phone to his ear, and I put the speaker on so we could both hear, and he prayed. And when he finished, my husband said ‘Amen’ right along with him, and that was the last word he said. And that was on a Friday night, and the following—not the next Saturday, but a week from that, is when he passed. But that was the last word he said—‘amen.’ After that prayer, he never said another word.”	“I was in denial … and disconnected about it. But … that's when I got my pastor involved.” “But when our pastor came and stood in the doorway and said, ‘P. peace be with you,’ it had an impact.”	(1) Pastors are the key to helping us understand prognosis and impending death. If the issues of prognosis arises, suggest that they may want to invite their pastors to the discussion of prognosis. Then ask the name of the pastor and say you would welcome them to the meeting.	(1) Pastors are the key to helping us understand prognosis and impending death. If the issues of prognosis arises, suggest that they may want to invite their pastors to the discussion of prognosis. Then ask the name of the pastor and say you would welcome them to the meeting.
(C) Church members as support	X	“My church family was absolutely my lifeline. They got me through everything because whatever N. went through, the church went through it, and it affected everybody in the church.” “I had a church family that was wonderful to me. They were absolutely wonderful to us.”	X	(1) Church members are a source of support for patients and family members. If patient and/or family members need support, ask whether a church member can assist. Ask for name of the church member and discuss how they can provide support.

PC, palliative care.

**Table 6.** Theme: Discussing Death and Prognosis

Sub-themes	Phase 1: focus groups		Phase 2: recommendations	
	AA	White	AA	White
(A) Death is not discussed	“And that was tough, because as a family unit we never discussed death. We never really sat around the dinner table and discussed death, and you know, really said, ‘well, this is what we're gonna’ do. We wanna’ make sure you're comfortable.’ … we just didn't talk about death, other than someone else's death.” “You know that [hospice] is the end of life, and I think that's because of our upbringing.” “As things progressed and he got more and more debilitated and weaker … and my concern was about him and did he have any fear about the end of his life. And so I never really did know how to approach him about it, but I just happened to be there one evening and we were talking, and he looked up at me and he said, ‘I'm not afraid to die,’ So when he said that to me, it gave me a calmness, a kind of peace within me.”	X	(1) Death is not discussed in our church or at home. Recognize that and approach this topic (death, impending death, possibility of death) with caution. (See section on prognosis for how to discuss prognosis.)	X
(B) Sharing prognosis in a negative manner	X	“But the way we found out was a nightmare, and I don't wish that on anybody, especially with a room full of people.” “All of these privacy things that everybody's so concerned about, and yet, at the same time, we have the entire waiting room being informed about your husband's, you know [health information] … And then earlier this afternoon, you [moderator] mentioned something again about HIPAA. So it's just—it's very frustrating that that's such a big deal and you have to sign these papers and all that stuff, and yet, when it's not convenient to the doctor, then [they disregard those procedures.]”	(1) Never share prognosis in a public space, and especially not in front of non-family members. (2) Never give date and time, always use range.	(1) Never share prognosis in a public space, and especially not in front of non-family members. (2) Never give date and time, always use range.
(C) Positive experience in sharing of prognosis	“The doctor explained it to me that the body is shutting down. He said, ‘You don't need to make him take him to the hospital and feed him, because the body is shutting down.’ He said, ‘Don't let them give you whatever to bring him back,’ he said, ‘because he is so frail, when they go down to press on him, they're gonna break the ribs.’ ” “I think when a person is understanding, then they're more accepting as to what is about to happen, and I think with him—both of us, it was comforting to know that we had that conversation … and it prepared us for what was gonna come.” “But Saturday morning, we went to see him and the doctor—one of the on-call doctors came by and he was telling him what was going on and how the lungs had really—were so compromised, that there was just nothing else they could do. So he, in his usual way, what is the prognosis. They with the military background, you know, he was always that. So the doctor was very honest. He says that is up to someone much higher than me, and I admire him for saying that because really, like he said, how do they know”	“The doctors were so caring and giving and hew was up one time and it was close to the end of life … and the doctors and the nurses were just crying with him and hugging him … just because he felt so bad and they were doing everything they could.” “The doctors at M hospital were wonderful. They told us, they confirmed that it was malignant, and then the doctor that was gonna’ do the surgery confirmed that it was very aggressive, but he said, ‘we can fight this together’ and he went on with encouragement … and the hope that you have to have… and they would get excited when we'd get a clear scan. So we all felt like it was a team effort and the doctor was working with us.”	(1) Explain in very simple and easy to understand terms. (2) Don't use medical language or terms. (3) Offer opportunity to ask questions. If family does not understand, explain it differently. (4) Physician responsibility to make sure he/she is clear and helps patient/family to understand.	As in (E) Who to share prognosis with, and how to do it
(D) Clinician specifying time to death	“I'm gonna touch on my grandfather because on a Friday afternoon, about—and the sky looked about the way it is now, we received—we—my mom and my sister and myself—received a phone call at the house that I'm staying in now, and that phone call told us that my grandfather had two and a half days to live. And I never, ever understood how a doctor can tell you two and a half days to live.” “and the doctor told me that they gave him three weeks to live. My husband lived three years after that. After three weeks, the VA doctor came to my house, the social worker came to my house, …. They came to see how my house was and if I was able to take care of him. … and after they checked and see everything and they had me to perform some things and they see that I was capable, they send him home. He stayed home about two years.”	X	X	X
(E) Who to share prognosis with, and how to do it	X	“I told the doctor, ‘don't tell her anything until you call me and tell me what the problem is.’ And when he did call and told me what it was, I says, ‘Please don't tell her. Let me tell her when she gets here,’ which would have been that day, but he did it anyways. So when they brought my mother to my house, she got out of the care, went straight to bed, and never got up again. And if he had listened to me that would not have happened. She gave up before she got here. So I really had some issues. It coulda’ been handled differently.” “My perspective is that … I had a good experience and I thought it was very loving, but the doctor saying it in front of the patient? Yes. Was that the right thing for my father? Yes. Would it be right for everyone? No, not necessarily.”	X	(1) Ask the family who to share the prognosis with. (2) Honor their decision. (3) Be a part of their journey.
(F) Miracles and hope	“As long as the patient is alive, there's always the possibility of a miracle.” “Only God knows when a person will die.”	X	(1) Ask family whether they want to know the prognosis. (2) Never be blunt. (3) Never tell the patient that they are dying. (4) Never put time and date on prognosis (always state as an estimated range). (5) Explain in VERY simple, non-medical terms what is happening in the body. (6) ALWAYS end by saying, “It's not in my hands; it's in God's hands.” If physician is not comfortable saying, “God” say, “It's in the hands of a Higher Power.”	X
(G) Bringing God into the sharing of prognosis	“So the doctor was very honest. He says ‘that is up to someone much higher than me.’ And I admire him for saying that because really, like he said, how do they know.” (God is the decision maker) “He had this wonderful doctor. All this man every talked about was God. That threw me off right there. I ain't never heard no doctor talking about God. Every time, you know, he's talking to me, he's saying something about God, and he said, ‘God got me her for this purpose, God got me here for this purpose, to make sure that he is well taken care of.’ And that is all he said.”	X	(1) If patient/family is religious, physician can say, “I see that you are a spiritual person. We are doing the best that we can and it's in God's hands.” (2) When sharing prognosis, always add that God is the decision maker, not the physician. IF not comfortable saying God, say “Higher Power.” for example, I don't decide, It is in God's hands OR if physician not comfortable, in the hands of a Higher Power. (3) If physician is comfortable, ask if you can pray with the patient/family.	X

**Table 7.** Theme: Family Caregiving for Patient

Sub-themes	Phase 1: focus groups		Phase 2: recommendations	
	AA	White	AA	White
(A) Take care of loved one at home	“And my sister, when she was in Florida and I was bring her home, she said, ‘Now, of anything happens to me, I don't want you to put me in no nursing home. Would you promise …?’ And I promise her. And I took her and she got worse and worse and worse. I took her just like a baby.” “I can remember the day that they released her from the hospital. Her main concern was not to go into a nursing home, and the family did abide by that, and I can remember that day. She'd kinda [leaned] me towards her and said, ‘I want you to go to home with me.’ And that's what I did. I was at her house between six and eight hours a day and once I week I would stay overnight.” “I think really what it boils down to is that you can't say we didn't take care of our loved one. We did. It's the way we was taught. We just take care of one another, so that's what we gotta come back to.”	X	(1) Recognize that members of an AA family take care of their loved ones themselves. It's a core value. (2) Don't bring up nursing homes as an option unless asked or unless patient is already resident of a nursing home, or about to be discharged to a nursing home per hospitalist orders. (3) If loved one is going to a nursing home, provide support to family.	X
(B) Take care of loved one at home no matter what the sacrifices	“It was my responsibility to take care of her. … the funny thing about it is that she got a hospital bed before she really got sick. I slept on an air mattress for 2 years. I own my own business so what happened is she would call me so I came up with the idea, that look, either I have to do my job or my wife, and I let the job go because I wanted to spend as much time as I could with her.” “So my uncle did not have to hear that [arguing about who will care for him]. Like oh, we—it's 6:00, where Jane at? I mean, I was Jane at 1:00, I was Jane at 3:00, I was Jane at 6:00, I was Jane at 8:00, I was Jane at 12:00, I was Jane at 1:00 in the morning. I was Jane. And I don't know, maybe I'm kinda on the crazy side. I didn't see that as a burden. I saw it as when I was a little girl, this man loved me. This man cared for me, and so it was my turn to give back. So he was not a burden to me.” “and I think it's taken us a while to get to where we allow other people to come into our homes and do that because we have always provided the care at all costs, and I don't think we realize that there are people that are trained to do that, so we don't have to be burdened—that we have our own family to get that care and we can take ourselves too. ‘Cause sometimes we're so busy caring for other people that we don't care of ourselves.”	X	(1) Don't raise issue or possibility of home “hospice” (see also section on hospice). Ask which family members are helping with caring for patient at home, and if so, what kind of help they are providing. If it is the kind of care home hospice provides, explain that this is the type of care that home health provides. (2) Stress that home health care is not there to take over. Stress that the family is in charge of making all decisions and determining how things are done. (3) Ask whether there are any concerns about the family providing the care that home health care provides (eg., cleaning a port, or bathing a patient who has an open wound); listen and discuss until all concerns are alleviated. (4) If, after this discussion, patient/family wants home health/hospice, ask whether they want you to recommend referral to this. (5) Stress that all decisions are up to patient/family. Stress that the PC physician is there to help, not to change way family takes care of loved one.	X
(C) Guilt at having a loved one in the nursing home	X	“Well, I went through the guilt thing too because my dad ended up, the last eight months of his life, he was at {nursing facility.] We just couldn't handle him at home anymore.” “So the whole time he was there, we had to pay … we didn't have to, but we paid a private caregiver to go in from 9pm to 6am every day to be with my dad through the night because we knew he was not gonna’ get any attention whatsoever there during the night. I mean, he was, our know, piss poor during the day … excuse my French … but, you know. At least he had some attention during the day. … And it breaks my heart.” “My mother was pretty sick and had to have care all the time and she was terrified of going into a nursing home because she knew that she wouldn't be cared for, and so, you know we struggled with … what to do.”	X	(1) Help family deal with guilt for putting loved one in nursing home.

**Table 8.** Theme: Hospice and Nursing Homes

Sub-theme(s)	Phase 1: focus group		Phase 2: recommendations	
	AA	White	AA	White
(A) Fears that hospice means death	“But when the hospice lady came, I would lock the gate. She called and I never let her in. She … called me on a Friday one day, she said, ‘I came to see G,’ I said, ‘Oh, we busy right now. I want you to come back in a few hours …. She said, ‘well, I'm here …’ I wouldn't let her. I just slammed the door. Yeah I did that for a long time.” “But we could never just say the word ‘hospice’ because hospice was death.”	“Hospice was a huge decision for me because if you sign for hospice, then you have to give up some things, like daily physical therapy… and the wrestling of having to make that decision ‘cos he was clear of mind the entire time and in pain… I didn't want him to give up hope.” “He stopped eating, and that was a big chore trying to get him to eat, but I—when I was aware that hospice was—that he was eligible for hospice, I started grieving then.”	See also section on Family Will Take Care of Loved One (1) Never mention the word “hospice” and do not raise the issue UNLESS the patient/caregiver raises the issue of hospice or expresses concern about burden of care OR asks about hospice. (2) Ask which family members are helping to take care of patient at home and if they are, what kind of care they are providing. If it is the kind of care home hospice provides, explain that this is the type of care that home hospice provides. (3) Ask whether there are any specific concerns (e.g., cleaning a port, bathing a patient with an open wound) about the family providing care, and discuss until all concerns are alleviated. (4) Make sure to emphasize that they are NOT there to take over; the family is the one who decides what and how it is done. (5) If open to it, talk about this is a helpful way to take care of the family at home. (6) Ask whether they have any concerns about this kind of home help. If yes, discuss until concerns are alleviated. (7) Whatever their response, acknowledge and respect their feelings/attitudes. (8) If, after this discussion, patient/family wants home health/hospice, ask whether they want you to make a recommendation for a referral to it. (9) Stress that all decisions are up to the patient/family. The PC physician is here to help, NOT to change the way family takes care of loved one.	(1) Assess how family and pt feel about hospice, but do NOT use the word “hospice.” Use “home health.” (2) Whatever their response, acknowledge and respect their feelings/attitudes. (3) If open to it, talk about how this is a helpful way to take care of the family at home. (4) Make sure to emphasize that this is an offer of help and assistance.
(B) Fears that hospice means taking change over the house	“It was like, well, maybe these people come in, talk about this man dying. I don't wanna’ hear this you know, so they don't come. That was my theory.” “Every time I look, there's a white person over this, there's a white person … look I gotta’ tell it like it is. Four of those people [from hospice] come to my house. All of them was Caucasian. Well, where are the black women? I find all of that … it really disturbed me. It really did. It got on my nerve.”	X	(1) Acknowledge and respect feelings. (2) Make sure to emphasize that this is an offer of help and assistance, but not taking charge or taking over. Explain that all decisions are ultimately up to the patient and family. “They are not there to change your home, your family, we are just her to say, ‘How can I help you?’ and then provide that help, and if the home help can't, then they will find out who can.”	X
(C) Our community needs to be educated about hospice	“But I had been educated about [hospice], then I could have, you know, I could have made better choices, and I would have been a nice person …. And I did apologize to them.” “Hospice … that was at the end of life. And I think its because of our upbringing, and I think if there was a way that we could educate … by means of seminars, or going into churches or whatever. If we could educate about what all hospice has to offer, and not see hospice as ‘they're coming, okay, they're gonna die.’ ”	X	(1) Recognize that rural AAs may not want to ask about hospice for a variety of reasons. (2) IF the patient/family do ask, explain about its services in the manner described earlier.	X
(D) Hospice support for patients	“And he was mouth open, everybody said they heard the death rattle … If he had a death rattle, I wouldn't have known. He wasn't eating anymore, and I said, ‘but he's hungry.’ She [hospice worker] said, ‘no, he's not hungry. He's not burning up no calories, not empty because he's not moving.’ She said. ‘And hospice, I love them.’ She said, ‘the body is shutting itself down,’ she said, ‘Mr. F. is not hungry.’ And I said, ‘but he ain't eating.’ She said, ‘mmm hmm.’ ” “So that's how hospice was …. I stand on the battlefield for them. They were just, … ‘what can I do?’ ‘what you need?’ ‘Do you need us to …’ Always treat you like you were the king of the hill like you were royal family. … I would never have enough to say about them ‘coz they played an important part in our lives. I told Mom, I said, ‘the minute anything happen to us, we going to hospice.’ ”	“And without her [hospice worker] help, and the last time N was in the hospital in Intensive Care, she met me, came that night, helped … and N was wanting a fan. I mean she raised holy heck with this nurse that said you did not need a fan, and she made sure he got a fan because he was burning up. So I mean, you know, the care that with the social workers and the nurses, they were awesome. I can't commend them enough, and they have been wonderful to me.” “[Hospice worker] was awesome at that. She also had the ability to give P a foot rub that would just calm him right down, and sometimes it was the case where we were upping the medication but the medication hadn't had time to take effect, we needed someone who could work a little miracle and buy us some time, and [hospice worker, bless her dear heart, could do … [Hospice worker] was awesome at that.”	(1) Those who received hospice care found it to be a source of support for patients.	(1) Those who received hospice care found it to be a source of support for patients.
(E) Hospice support for caregivers	“A lot of things were explained to me, and I thank God for that agency and the people who came into our lives were there, because if they weren't there, I wouldn't even know what to do. I wouldn't even know my head from my toes.” “They [hospice workers] wanna make sure not only the person that you're giving care to is being well taken care, but they want you to be well—and I don't know what it is, but seemed like you just—even the care with hospice people, seemed like you automatically became family. It wasn't like you were inviting some strange person that you never seen before when they come to your house, you know, like—it was none of that. They talked to my uncle like they knew him all his life. They talked to me, and I thanked God for hospice because if it was not for them, I wouldn't have a clue.” “But hospice—they helped—they was there for my mom, but it turned out, it ended up they were more there for me.”	“And I can't say anything but just accolades for them because I could not have done it, and I knew when my—when A. (Hospice Worker) was there, I could walk outta the house and I did not have to worry one minute.” “And so, you know, as I said, all the people that came into our house, even other certified nursing assistants, I was very pleased with them because they listened to what I was telling them.” “We had a social worker come in and spend a lot of time with us and they called several times. We were very impressed with the hospice that were involved with us.”	(1) Those who received hospice care found it to be a source of support for caregivers.	(1) Those who received hospice care found it to be a source of support for caregivers.
(F) Hospice care support by chaplain	X	“They were there every day, you know, and the chaplain stayed with me, and I did not need bereavement after. I mean, you know, it's very difficult, but they were there for me and I didn't need the bereavement group, but they had a memorial and I did go and they were so kind, even that night, to me and everybody else.” “The chaplain from the hospice that we, you know, he said he knew I wouldn't come … ‘cause she was taking … and he said you think she wants to talk to me. I said I will give her your number if she does. They reached out—they reached out to our family in New York for me, which …, but they did.”	X	Those who received hospice care received support from chaplain.
(G) Hospice care support after death of loved one	X	“[I] was holding him when he died, and when they came to get him, she—the hospice nurse—took me into the dining room and said let's sit down and just—do you wanna pray. And I said yes, but she wouldn't let me watch.” “The hospice nurse was there. We were changing the sheets when my husband started to pass, so she left me alone with my husband. She called the funeral home, she made the arrangements to have him picked up, and she stayed after he left, and she got everything out of the house. There wasn't a bedpan, there wasn't a bedside commode, there wasn't bed rails. He never had a hospital bed. He went very quickly, but the shower chair was gone, just everything that reminded me when I walked in our bedroom of it being a sick room. She took—and most of it, you know, of course, was donated to the hospice to be used again.” “The morning that he passed away, I had so much family there, children, brothers, the house was packed full. Everybody was sleeping anywhere they could find to sleep, but when it happened, we were all numb. There was so much emotion going on, and the hospice nurse, the head one, took over everything. All we had to do was just sit there ‘cause we were numb. She took over the whole thing. Everybody was called, taken care of, she got everything out of the house, and she stayed. She was wonderful. That's a hard—I could not have done whatever needed to be done. I could not have done that.”	X	(1) Those who received hospice care found it to be a source of support after the death of a loved one.

pt, Patient.

**Table 9.** Theme: Clarity About Opiate Dosage

Sub-theme(s)	Phase 1: focus groups		Phase 2: recommendations	
	AA	White	AA	White
(A) Lack of clarity about medication and medication regimen administration	“I could not understand why the medical field, they know a person is dying, yet they coming up with some kind of medication that they wanna' put in her mouth you know. So I told the head nurse, I said, ‘Well, why would you guys give here this medication and she's dying?’ She had stopped talking, she had stopped eating, all her body function was, you know, deteriorating okay, and this is what they told me, and I didn't like it at all. They told me that the medication that they was giving here was to ease her pain. I said, ‘she can't fell no pain. She's dying you know, and that's the problem. You know, that really got to me.’ ” “but that Tuesday, the nurse called in sick and they got a temporary nurse, and she gave him too much medicine ‘cause he told me that he was in la-la land. And then his daughter called and he told his daughter and his daughter called and got on the administrator of hospice and they came down and they apologized and that nurse that gave him too much medicine, they made her check on him every five minutes.”	“The pain had gotten pretty bad with that pancreatic cancer, and the nurses and the medicine bottle told us how much to give, and they also specified, ‘just give this’ but they would come in to check on him, they would say, ‘well, you can give him more.’ We always got confused. Can we give him more, or do we have to follow what's on the bottle? And that was always an issue, even until the end, we never knew what the guidelines were on that.” “The doctor [would not give her pain medicine]; he was worried, that she was gonna’ get addicted, dependent, that's dangerous, You know, you have to tell the doctor, ‘look, the pain … give her the pain medicine … She's 96 years old … Please.’ ”	X	(1) Explain what each medication is for in simple, easy to understand terms, especially the administration of morphine and its dosing.

**Table 10.** Theme: Advanced Care Planning

Sub-theme(s)	Phase 1: focus groups		Phase 2: recommendations	
	AA	White	AA	White
(A) Advanced care directive/DNR in writing	“We didn't have any words written out you know, we didn't have anything to really follow. It's interesting, it just happened. As a family, we are all accepting of … how that went.” “As a family unit, we just had to know in our hearts that this was the right thing to do, this is what he would have wanted. We didn't have an … Outline of what we needed to do next.”	“We were in the ICU, he said, ‘This is the last time I'm coming to the hospital’. He said, ‘I will not come back’. And he signed the DNR. He told our social worker and the nurses, ‘You do not bring me back to the hospital. Everything is signed.’ He begged us not to let him die at the hospital … And whatever F. wanted, we put into place, and we did our best to have it. And he went out just like he wanted. He didn't wanna’ be resuscitated, no kind of life support, anything … And when he made the decision to stop dialysis, he know with his body like it was, the max he would have would be four days. And he made it three and a half.” “He had a DNR … but he was at home. Now nine days or so before he died, our hospice had just gotten a new doctor, and the new doctor wanted to talk about feeding tubes, and …. The hospice nurse was there, and she said, ‘No, that's not what he wants.’ But he [doctor] insisted on talking to T. and T. said, ‘That's not what I want.’ But the doctor wanted to consider it.”	(1) Don't ask whether they have an ACP document. (2) Ask whether loved one shared instructions/directions of what they wanted with a family member. If yes, ask whether you can speak with that family member. (3) Ask family member what patient wanted and follow those requests.	(1) Ask whether they have any document of the patient's wishes in writing (don't specify which kind). (2) If they have a written document, ask what these specified and ask whether the patient has the same wishes or whether they have changed, and how are these being followed in the hospital. (3) If patient does not have AD, ask whether they know what the patient wanted in terms of care. What these specified and whether the patient has changed wishes/same wishes and how they are being implemented.
(B) Confusion between advanced directives and power of attorney	X	“Well, I had gotten the advance directives from the hospital social worker, but … I probably didn't have a clear understanding. I always thought I had power of attorney because in the bank I had power of attorney, coz’ I could write, you know, whatever that was, but then when it came down to the insurance and all those other things, they were like, ‘you're not really power of attorney.’ ” “I had health care power of attorney, but I didn't know that that wasn't power of attorney until it was too late.”	X	(1) Ask whether they have been asked to complete any documents and whether they do, do they have any questions about any of these. (2) If they do, clarify very simply. (3) If patient does not have an AD, ask whether they would like to complete one.

ACP, Advanced Care Planning; AD, Advance Directive; DNR, do not resuscitate; ICU, intensive care unit.

**Table 11.** Theme: Need for Services

Sub-theme(s)	Phase 1: focus groups		Phase 2: recommendations	
	AA	White	AA	White
(A) Need for specialized services for military personnel	“Now my husband … was a military man, and … very private person and it took a lot for anybody to come in and bathe him and change him. We would go to the VA hospital and they would always tell him, ‘okay, you served your country. You have earned the right to have home health, just home health care come in and help your wife’. My husband always said ‘no, we okay.’ And that's how the conversation went … I mean I took care of him for seven years, and then the last three years when he couldn't even walk anymore and we'd go back and forth to the hospital, ‘do you want care?’ ‘No, we okay. We fine.’ And this went on until April of this year, and then he allowed home health to come in couple days a week and they were able to, you know, bathe him. He accepted that. He saw—I think he saw that I was getting tired, and so he allowed them to come in and bathe him.” “My dad was a very strong man. My dad was a Marine. [The] mentality that they're very strong and, you know, they endure to the end and it was initially difficult for my dad to come to the—face the fact that we would be taking care of him ‘cause he's always taken care of us. I mean we are grown with children, grandchildren, my dad used to [take care of us]. And so it was really difficult for him to come to terms with us caring for him. And we just tried to impact upon him how fortunate we thought that we were ‘cause we were able to return some service at the end that he had given us.”	“Being from a military background, we had—when we had an aide come in near the end, we had to say do not cover his feet, and then, of course, thankfully, they went with that.” “And again, if you have someone with a military background that has been in combat, they're going to, you know, maybe do things like, you know, I don't care how cold it was, there was at least one of … feet out at all times, but other little quirks or things that they learned and they listened. And for that reason, I was very comfortable.”	(1) Understand someone who is from a military background.	(1) Understand someone who is from a military background.
(B) Need for specialized services for southern men	X	“My husband saw the chaplain from hospice and a priest, but my husband was a very southern man, and he was very internal. He didn't share feelings. You didn't do that when he was growing up, and you were a man in the south. You didn't cry, you didn't—not that he wasn't loving and caring with his family, but he wasn't going to burden anyone with his issues. And so, you know, they offered. They sent in social workers and—but, you know what I mean by southern man.” “The fact that his head RN had—was retired Naval petty officer, I'm here to tell you the man was not much taller than me, and I don't think he had an extra calorie on his body, and as I said to … one time, I said he was not serving in a hospital; he was out in combat… But there was a very close bond between him being the head RN and … you know, and I don't know how to explain that at all other than saying it was essentially one military, you know, sort of man with another.”	X	X
(C) Need for financial assistance for those in financial need	“Low Country Council of Governments will give you up to $500 worth of stuff, so if you go home and see that you need to change your plugs to a three-prong plug-in outlet, they'll get a man to come in there and they'll pay for them, and that'll get your house set up, you know, okay so that you can live, but it's right here in the community, but it's just like no one really knows. No one tells anyone. So hopefully we can all find out and just let some people know.” “and then if you find out about it, you gotta go through so much. You got to have a certain income. You got the wait on this waiting list. You got to go through so much. It shouldn't be like that. Everybody should be to get this ___, you know. That's the way everybody want, to take care of their own.”	“Well, nobody said … ‘you need to apply to Social Security for extra help.’ That's what it's called is extra help … And it would have been nice if I didn't have to spend three weeks, because I didn't know what I was doing. I was navigating blindly. Surely somebody out there knew… that this thing existed …” “I went to the Good Neighbor Clinic when … first got really, really sick because we could not afford some of his medications … But anyway, I swallowed my pride and I went to the Good Neighbor Clinic and I begged, borrowed and pleaded … and said ‘look, my husband is going to die if I don't get some help. And I mean you all are here to help us.’ … luckily I had a case worker there that helped me and she got on the computer and she filled out all the forms … and we ended up getting most of them for free, and then we got turned down on some.”	(1) AA community do not ask and do not know what is available. There is a need for community members to be aware of community resources.	X

Lack of trust in the health care system and in physicians was a theme in the AA group (Table 2). To build trust, CAG members suggested that they meet and greet AA patients and families first, introduce themselves, briefly describe the study and the consult, and explain that they were an integral part of the development of the protocol. Although the issue of lack of trust did not arise among White CAG members, they felt this would be beneficial for White patients too, and suggested they be the first to meet White patients and families.

To do this, CAG members had to become hospital volunteers and undergo volunteer training. All but one CAG members did so. The CAG also developed a colorful page about the study in which they used images of the ethnically diverse CAG working on the study, a photograph of the PC physician for this study, a computer with a person in a bed talking to a patient, and a simplified illustration of the data to be collected, which they would share with the patient and family.

Telehealth, the remote delivery of health care and sharing of medical knowledge using telecommunication, has been used to deliver health care to remote areas,^78–84^ and it was the method by which this consult was conducted primarily due to the health professionals' shortage (including PC services) in the rural South, together with large geographical distances in rural areas. Since this method was not widely used in local health care settings, we anticipated that community members might be unfamiliar with it. We therefore, raised the issue in the focus groups to hear how community members felt about it and what their suggestions were for a reduction in potential community concern. Recommendations made to overcome this discomfort were the same for both ethnic groups and included the PC physician wearing a white coat (to indicate that he/she is a physician), as well as the PC physician acknowledging the inability to be physically close to one another. They also requested that a family member be in the room, and, to ensure continuity of care between the tele-consult and hospitalists, that the study coordinator be present (Table 3).

A theme that arose in the White focus group was their experiences of physicians acting with a lack of sensitivity, outright rudeness, and/or not respecting confidentiality (Table 4). Although this was not raised by the AA patients, both ethnic groups strongly endorsed the recommendation that the PC physician conducting the consult would always treat the family and patient courteously, and never violate patient confidentiality.

Table 5 highlights a significant cultural value that impacts both ethnic groups, the role of religion and church in the lives of both ethnic groups. Although religion and church was important to both groups, in the AA group, religion was considered the source of all comfort, with the church serving as the center of all aspects of community and personal life. The pastor played a key role in helping family members accept the reality of the impending death in both groups. A difference occurred in terms of support: In the White group, it was the church members that provided support; whereas in the AA community, family was key (as seen in Table 7).

Table 6 highlights a core cultural value of the AA group, and that is that the family takes care of their loved one, regardless of the sacrifices it requires. “It's the way we was taught. We just take care of one another.” Sending a loved one to a nursing home was considered unacceptable among the AA group, and only one CAG member had a loved one in a nursing home, due to the patient having Alzheimer's and continually running away, requiring her to be in an enclosed environment. This has implications for the physician referring patients to home hospice or nursing homes (see also Table 8) where nurses or other professionals will come into the home.

Three themes about death and dying emerged in the AA focus group, all of which are tied to church teachings and doctrine. Death and impending death were not talked or preached about in church, and consequently, not discussed in community members' homes; the concept of maintaining hope, a fundamental aspect of faith, was reflected in names of AA churches, in pastoral messages, and in community songs, and the unshakeable belief that whatever happens to the patient is in God's hands.

Based on these concepts, the CAG asked physicians to understand these concepts, recognize them as important, approach the concept of death and dying with great caution, and explain that the decision was in “God's hands.” If the physician was uncomfortable with “God,” CAG requested that the physicians say, “It's in the hands of a higher power.”

Specific guidelines for sharing prognosis with AAs included never telling the patient that he/she is dying, under no circumstances specifying time until death, explaining what is taking place in the patient's body in non-medical terms, and always stating that the decision lies “in God's hands.” Sharing the prognosis in an insensitive, disrespectful manner or sharing it with a patient when the caregiver asks for it not to be shared by the physician were concerns raised in the White focus groups. Recommendations, therefore, included physicians respecting privacy and not sharing prognosis in a public space and/or in front of non-family members, and asking family whether they wanted to know the prognosis and then respecting their choice.

Although the word “hospice” was associated with death in both ethnic groups, in the AA group it also raised fears of strangers, White people coming into their home, and taking over. There was recognition that the AA community could benefit with education about hospice and what it offered. The AAs that had used hospice services in the care of their loved ones found some of its services, especially the support of the patient and caregiver, very beneficial. In addition to these two areas of support, the White group reported receiving support by the hospice chaplain, and support by hospice staff after the death of their loved ones. In the AA group, support was provided by the family church's pastor and family members.

Because of the concerns in both groups about the association of hospice, CAG recommendations included not using the word “hospice.” In the AA group, the physicians were asked not to raise the issue of home care unless the family asked for assistance and even then, were requested to provide reassurance that the hospice staff are not there to take over, and the family will remain in charge of taking care of their loved ones (Table 9).

Confusion between the various documents such as Advanced Care Planning, Advance Directive, Health care Power of Attorney, and Do Not Resuscitate occurred in both groups. Although only a few White focus group members had an Advanced Directive, several had seen or been asked about one. This was not the case in the AA group; here was an expressed understanding of knowing what the loved one wanted in terms of care, because the loved one had often shared it verbally with a family member, and that family member wanted to do whatever the loved one had expressed (Table 10).

An issue of concern that arose among members of both ethnic groups was lack of clarity about the administration of morphine, especially the dosage since that did not always follow usual medication regimens, as well as the possibility of lack of consciousness, and even death. The CAG members therefore made recommendations that would clearly explain this to the caregivers.

Table 11 indicates the perceived lack of needed services for those living in the rural South, such as need for specialized services for military personnel. Although the need for financial assistance for those in need was present in both groups, it was clear that in the AA group there was a lack of awareness about services that were already available in the community. This inspired the AA CAG members to create a booklet describing available services, designed specifically for older AAs, using large font and photographs of AAs using these services. These booklets were widely distributed in the AA community.

Table 12 summarizes all the recommendations for the culturally based PC consult, and it is presented side by side with the National Palliative Care Guidelines for a PC consult.^20^ It includes all the themes and clearly specifies which culturally based aspects are relevant to White or AA patients and families or to both groups. This is the guiding protocol that was used by the PC physician (as well as the study coordinator) to guide the PC consult in Phase 3.

**Table 12.** Consult Guidelines: Culturally Based Compared to National Consensus Project Guidelines

NCP guidelines^a^	Culturally Based Guidelines	
	AA	White
	Understand distrust (AA)	
	Lack of trust of medical system and care. Recognize and respect that there are historical reasons for this. Work to establish trust.	
	Reduce distrust: AA patients/caregivers are more likely to trust AA members of their community. (1) All AA patients/caregivers will first meet an AA CAG member who will introduce them to the study (but not review consent). (2) If patient/family agree, CAG member will introduce them to the Study Coordinator who will review the consent.	Although lack of trust was not a concern, White patients and caregivers will first meet a CAG member (W or AA) who will introduce them to the study (but not review consent).
	Enhance trust and address telehealth	
	(1) PC physician is not in same facility, pt/family need to have some indication that he/she is a clinician (doctor.) Wear White coat.	
	(2) Meeting patient/family via telehealth, acknowledge at the beginning of the session that this is not the same as sitting next to one another.	
(d) A thorough review of: (i) medical records; (ii) relevant lab results		
(e) A review of: (i) medical history; (ii) therapies; (iii) recommended treatments; and (iv) prognosis		
(f) Identification of: (i) comorbid medical; (ii) cognitive; and (iii) psychiatric disorders		
(g) A medication reconciliation including over-the-counter meds		
	Address patient and family:	
	Do not call patient by first name unless invited to do so.	
	Never be rude, always be courteous; Always respect patient confidentiality and never share prognosis in a public space, and not in front of non-family members.	
	(1) Introduce self (PC physician), then ask patient and caregiver and all else in room to introduce selves. Hospital staff and study coordinator last.	
	(2) Acknowledge telehealth medium.	
	Establish rapport:	
	Get to know the patient, establish rapport	
	Take and make time to get to know the patient and the family.	
	Learn something about the patient's family, for example, patient's past occupation, where he/she has lived. Repeat it back and converse about it.	
	Bring up something local, (e.g., About local geography, local history) to indicate that you know about the area.	
(h) Social determinants of health, including: (i) Financial vulnerability, housing, nutrition, safety.	Recognize financial vulnerability:	
	There are many in the AA community and some in the White Community who experience financial hardship. Recognize many experience substantial financial difficulties and the realities this brings. For example, have realistic expectations; recognize that some things that we may take for granted, for example, having A/C is not available for all.	
	AA community do not ask and do not know what is available. There is a need for community members to be aware of community resources. (The group developed a brochure specifically aimed at AA to bring awareness of services to AAs. Used AA visuals and large font).	
(j) Patient and family emotional and spiritual concerns, including previous exposure to trauma	Understand role of religion and church:	
	Pastors are the key to helping us understand prognosis and impending death. If prognosis is to be discussed, suggest that they may want to invite their pastors to the discussion of prognosis. Then ask name of pastor and tell them you would welcome them to the meeting.	
	Religion is the source of all comfort, a key value, and it is the perspective from which AAs view the world. Therefore, in all PC physician interactions with AA patients recognize and respect that this is an INTEGRAL part of all that is said and done.	Church members are a source of support for patients and family members. If patient and/or family members need support, ask whether a church member can assist. Then ask for name of church member and discuss how they can provide support.
(l) Patient and family needs related to: (i) anticipatory grief; (ii) loss and bereavement including assessment of family risk for prolonged grief disorder	Understand death and dying (AA)	
	Death is not discussed in AA church, nor in our homes. Recognize that and approach this topic (death, impending death, possibility of death) with caution.	
	No AA person dies alone. If they have no one, a pastor will come and sit with them so that they are not alone during the transition.	
(b) Determination of (i) decision-making capacity OR (ii) identification of the person with legal decision-making authority	Understand family will take care of loved one (AA)	
(i) Social and cultural factors and caregiving support including: (i) caregiver willingness and capacity to meet patient needs	AA families take care of their loved ones themselves in their homes. Even if there is sacrifice, one or other family member will always be there to care for loved one.	
(k) The ability of the patient, family, and care providers to: (i) communicate with one another effectively: consideration of language, literacy, hearing, and cultural norms	See also: Understanding death and dying	
(a) Patient and family understanding of: (i) serious illness	Understand talking about prognosis	
	(1) Ask patient/family whether they want to know prognosis.	(1) Sensitively determine whether patient/family want to know about prognosis.
	(2) Never be blunt.	(2) Honor their decision (i.e., if don't want to know, don't discuss and vice versa).
	(3) Never tell patient they are dying.	(3) Be a part of their journey.
	(4) If family asks prognosis, give it in range only (never give date or time).	
	(5) Explain reasons for what is happening in the body very simply (and do not use any medical terms).	
	(6) Offer opportunity for patient and family to ask questions. If family does not understand, explain it differently. It is the physician's responsibility to make sure he/she is clear and helps patient/family to understand.	
	(7) If patient/family is religious (highly likely), physician can say, “I can see that you're a spiritual person, we're doing the best that we can and it's in God's hands.”	
	(8) Always add that it is in God's hands/God decides. If physician is not comfortable saying, “God,” say, “it's in the hands of a higher power.”	
	(9) If physician is comfortable, ask whether you can pray with the patient/family.	
(a) Patient and family understanding of: (i) goals of care, (ii) treatment preferences, and (iii) AD if available.	Understand goals of care, treatment preferences, and ACD	
	(1) When discussing Advance Care Directive, many patients/family confuse this with Power of Attorney, DNR, and will. Ask what documents (if any) they have.	
	Recognize that Care instructions are given verbally to family member(s). There is very low likelihood of ACD but may have DNR and will.	(1) Ask whether patient had any document of patient wishes in writing (don't specify which kind.)
	(1) If patient is unable to communicate: Ask if loved one shared instructions/directions of what they wanted for care with a family member. Ask who the family member is.	(2) Ask whether they have been asked to complete any documents. If they have any questions about these, clarify very simply.
	(2) When PC doc speaks to family member, ask what the patient wanted in terms of care.	(3) If they have a written document, ask what these specified and ask whether the patient has the same wishes or whether they have changed, and how are these being followed in the hospital.
		(4) If patient does not have AD, ask whether they know what patient wanted in terms of care.
		(5) If patient has no AD, ask whether they would like to complete one.
Post-discharge plans	Understand perceptions of hospice	
	See also: family will take care of loved one	
	(1) Never mention the word “hospice” and do not raise the issue UNLESS the patient/caregiver raises the issue of hospice or expresses concern about burden of care OR asks about hospice.	(1) Assess how patient and family feel about hospice but do not use the word, “hospice.” Use “home health.”
	(2) Ask which family members are helping to take care of patient at home and if they are, what kind of care they are providing. If it is the kind of care home hospice provides, explain that this is the type of care that home health provides.	(2) Whatever their response, acknowledge and respect their feelings/attitudes.
	(3) Ask whether there are any specific concerns (e.g., cleaning a port, bathing a patient with an open wound) about the family providing care, and discuss until all concerns are alleviated.	(3) If open to it, talk about this as a helpful way to take care of the family at home.
	(4) Make sure to emphasize that they are NOT there to take over; the family is the one who decides what and how it is done.	(4) Make sure to emphasize that this is an offer of help and assistance.
	(5) If open to it, talk about this as a helpful way to take care of the family at home.	
	(6) Ask whether they have any concerns about this kind of home help. If yes, discuss until concerns are alleviated.	
	(7) Whatever their response, acknowledge and respect their feelings/attitudes.	
	(8) If, after this discussion, patient/family wants home health/hospice, ask whether they want you to make a recommendation for a referral to it.	
	(9) Stress that all decisions are up to the patient/family. The PC physician is here to help, NOT to change the way family takes care of loved one.	
	Understand perceptions of nursing homes	
	(1) If patient is in the nursing home, or family/patient brings it up, PC doc can discuss nursing home referral. If not, do not raise it.	If patient is in nursing home, help family deal with guilt about needing to place loved one in nursing home.
	(2) If loved one is going to nursing home, provide support to family.	
(c) Physical examination/ASK about: (i) identification of current symptoms; (ii) functional status	Explanation of medications	
	(1) Explain why pain medication is needed, especially the administration of morphine and its dosing (and why it varies and more may be administered than family expect.	
	(2) If there is concern about lack of consciousness raised, explain balance between lack of pain and lack of consciousness.	
	(3) If concern about getting more morphine than was originally scheduled is raised, explain dose is flexible based on patient response.	
	(4) If concern about addiction is raised, explain that addiction is not an issue and why not.	
	(5) If fear of overdosing is raised (with potential to enhance death), address concern and ease fear.	
	(6) Explain clearly, simply in non-medical language.	

^a^ Numbering listed per NCP guidelines.

ACD, Advance Care Directives; W, White.

### Phase 3: implementation

**FIG. 3.**
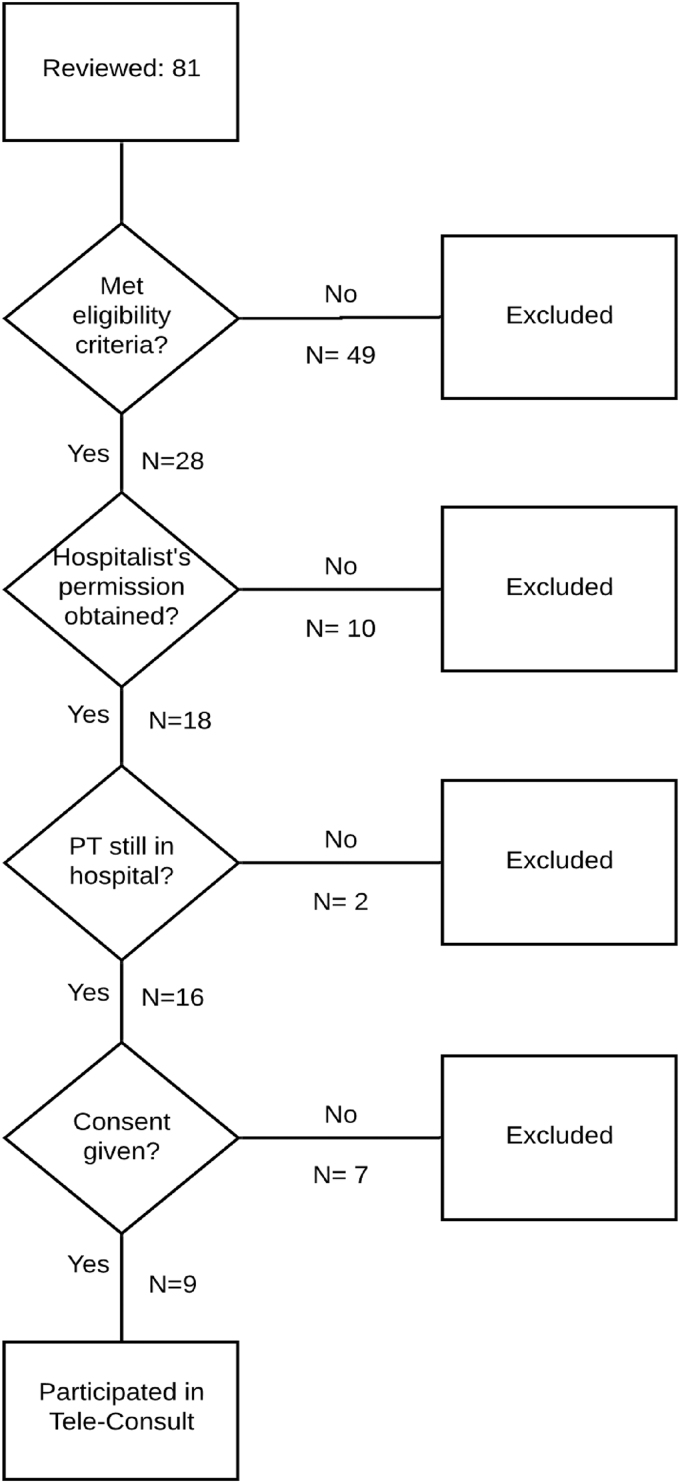
Protocol implementation.

Phase 3 Study Flow is illustrated in Figure 3.

Although 6 months were originally allotted for Phase 3, reluctantly we had to half this to 3 months as a result of the unexpected delay before the PC physician, who is from another state, received his SC medical license. (At the time of study implementation, there were no PC physicians in SC.)

### Program implementation: structure-process-outcome

#### Structure

(1) Support from Hospital Leadership was high as indicated by permitting the study to be conducted at the BMH and by receiving IRB approval from the hospital IRB; and (2) referral by hospitalists: only 18 out of 28 eligible patients were referred to the study by hospitalists.

When several calls from the Study Coordinator to hospitalists informing them of an eligible patient were either not returned and/or a consult was not prescribed, we identified this as a barrier. After a discussion between the PC physician and the chief hospitalist, the protocol was changed. The hospitalist granted permission to text them directly to let them know a patient was eligible, requesting that they opt out if they did not want to patient to be included. After this protocol revision, (a) there was an increase in referrals (in the 14 weeks pre-protocol change, there were 8 referrals [$\bar{X}$:0.58/week] and in the 8 weeks after the change there were 11 referrals [$\bar{X}$: 1.36/week]); (b) calls not returned by the hospitalists dropped from 4 to 0; and (c) eligible patients not allowed to be approached for study participation remained the same (4 in 14 weeks pre-change [$\bar{X}$: 0.28] and 2 in 8 weeks post-change [$\bar{X}$: 0.24]).

#### Process

(1) Implementation fidelity: adherence to the study protocol by the PC Physician was determined based on a checklist of 30 protocol-determined items that were to be adhered to during the tele-PC consult. The PC physician scored 30 out of 30 for six consults and missed one item only on three consults (once was not acknowledging the telemedicine, and twice not asking the patient whether they would like to speak to a pastor). (2) Follow through by the patient's physician on the PC physician's recommendations: The PC physician made specific recommendations for patient care in four instances (in five other cases there were no additional recommendations in addition to the care the patient was already receiving). Hospitalists followed recommendations in three of the four instances.

#### Outcome

(1) Participation rate: All 16 eligible patients approached were too ill or non-communicative and unable to provide consent; therefore, family caregivers were invited to participate in the study. Nine caregivers (six AA and three White) consented, and seven declined (three AA and four White), that is, the reach was 9 out of 16 (56%). (2) Family satisfaction was assessed by using the FAMCARE-2 (family members)^85^ designed to assess family member satisfaction with care received by PC teams. This 17-item instrument is designed to measure family satisfaction with a PC program; since this was only a consult, many of the questions were not applicable (e.g., speed with which symptoms were treated, way in which physical needs for comfort were met, etc.). We therefore present responses to the 10 questions that were applicable (Table 13). Patient satisfaction was not assessed, as almost all the patients were non-verbal or non-communicative during their hospitalization. In all the applicable questions, there was a high degree of satisfaction (either satisfied or very satisfied) with the PC consult. Additional comments made by family members included “He helped her to respect herself”; “How can I say it? He is just wonderful, I am so thankful”; and “talking with him gave us peace of mind.”

**Table 13.** Modified FAMCARE-2: Selected Questions

Q		No.	S	VS
2	The way in which the medical condition and likely progress were explained by the palliative care doctor in the consult(s)	8	2	6
3	The way in which the palliative care doctor respected the dignity of the patient and family in the consult(s)	6	0	6
4	Consults with the palliative care doctor to discuss the patient's (your) medical condition and plan of care	8	1	7
6	The palliative care doctor's attention to the patient's (your) description of symptoms during the consult(s)	7	1	6
8	Availability of the palliative care doctor to the family	7	0	7
9	Emotional support provided to family members by the palliative care doctor during the consult(s)	7	0	7
10	The practical assistance that the palliative care doctor referred the patient (you) to	5	1	4
11	The palliative care doctor's attention to the patient's (your) symptoms during the consult(s)	7	1	6
12	The way in which the palliative care doctor included the family in treatment and care decisions during the consult(s)	7	1	6
13	Information given by the palliative care doctor about how to manage the patient's (your) symptoms during the consult(s)	5	0	5

S, satisfied; VS, very satisfied.

## Discussion

The aim of this study was to develop a culturally based PC tele-consult for and by rural, southern AA, and White communities and to test the feasibility of implementation. The program was feasible to both develop and implement. The main implementation challenge was the lower than expected referral by hospitalists. Although this improved after a change in protocol in which they were texted when a patient was eligible, it is clear that getting full buy-in from hospitalists, in addition to leadership and nurses, is essential in conducting hospital-based studies in small, rural hospitals.

This study is the first in the United States to have developed an ethnic-group specific, culturally based palliative consult intervention by and for rural, southern White and Africans elders with serious illness. In contrast to the existing model of end-of-life care that is based on White, middle class cultural and religious values,^1–3^ this model was designed to address the unique cultural values, beliefs, frame of reference, and preferences for communication and care of each ethnic group.

Differences between the two groups were evident in most of the themes (8 of the 10). Based on their ethnic group's themes, community members from that group specified ethnic-group-appropriate communication strategies, later incorporated into an ethnic-group-specific protocol, which the consultant used when conducting PC consults.

Two key cultural values in caring for a loved one with a serious illness emerged: (1) the integral role of faith, religion, and church; and (2) the role of family in caregiving, with both similarities and variations between the two ethnic groups. The South is commonly referred to as the land of the Bible belt, a place where the population is profoundly religious, with widespread religious practice and deeply held beliefs^86^; however, the separation between the White and AA church that began with the creation of separate AA churches in the 1880s after the Civil War remains in effect today.^87^

Although both groups reported on the importance of religion and church, for the AA community the church was considered the source of all comfort and learning, and the center of community life, spiritually, socially, and culturally, a finding widely acknowledged by Southern religious study experts^87^ who consider the church as the most important institution in the AA culture.^88^ In both ethnic groups, it was the pastor who played a key role in helping the patient and family accept impending death.

In the AA community, the pastor is traditionally considered the leader of the community, to whom community members look for guidance.^89^ The need to have the presence of a spiritual leader actively present to participate in treatment decision making and in discussing end-of-life issues with the patient and families has been reported in a prior study.^56^ “Living your life in faith, each and every day,” as expressed by the AA pastor on this study's CAG highlights the key role of faith and trust in the power of God in the AA community, at all times, especially during the final stages of life. A similar finding was described by Johnson et al.^90^ in which three aspects of faith during the dying process were described by an AA participant: (1) trust in the power of God to take care of the person, (2) recognition that death is the beginning of a new life, and (3) belief in a better life with Jesus after death. Based on this faith, AA CAG members highlighted the importance of the clinician saying, “It's in God's hands” when discussing prognosis with an AA patient and/or family members.

“It's the way we was taught. We just take care of one another,” as explained by a CAG member, highlights another core value in the AA group. In her review of the caregiving field, comparing AAs and Whites, Dilworth-Anderson et al., 2002, found that experiences and outcomes of caregiving varied across racial and ethnic groups.^91^

Reasons for providing care to older relatives also differed between the groups, leading to the conclusion that cultural values and beliefs serve as the lens through which caregiving is experienced.^92–94^ Suggested reasons for the high value that AAs place on care of family members are rooted in history where no outside care was available, and family networks served as systems of social service, welfare, and community-based interventions.^95^

### Study strengths and limitations

Relying on CBPR as our study's guiding principle, with community members from each ethnic groups being integral to all phases of the study, resulted in the shaping of the study in ways that enhanced the study. The unequivocal recommendations by the AA members of the CAG to conduct separate focus groups (Phase 1) permitted a detailed comparison between the two groups. The ethnic-group-specific recommendations for the tele-PC consult (Phase 2) resulted in the creation of first ethnic-group-specific PC consult protocol; and in Phase 3 the initial introduction of the study to patients and family by CAG members who had created and shaped the consult to meet the cultural values of each group was warmly received by patients and family in both groups.

The dedication of the CAG members to the lengthy commitment (program design required monthly meetings over 28 months) was remarkable; only 1 of the 12 CAG members missed a meeting (a recent widow, her marriage to a widower overlapped with a meeting). The CBPR process also resulted in a strong sense of both community's commitment to, and ownership of, the process, as well as the development of friendships among CAG members.^96^

This study has several limitations. First, it took part in one site in one southern rural state, and only focused on White and AA communities, therefore limiting generalizability beyond the rural south or to other ethnic groups. The period for testing the culturally based protocol (Phase 3) was shorter than anticipated and limited the testing of the short-term efficacy of this model.

However, the effectiveness of this model is now being tested in a randomized clinical trial (RCT; R01NR017181) in rural hospitals in three Southern states (Mississippi, Alabama, and South Carolina) in which this culturally based program, in addition to standard care, is being compared with standard care alone. Additional outcome measures are included in this trial—patient symptom burden, patient and caregiver quality of life, family satisfaction with PC, patient feeling heard and understood, caregiver burden, and resource utilization. Since referral of patients by hospitalists was the main challenge in this study, in the RCT we have partnered with hospitalists at each of the three rural sites. Beaufort CAG members play a critical role in the trial; they have trained the four PC physicians who will be using the culturally based PC intervention and have provided guidance and inspiration to the CAG members at each of the three participating sites.

### Health equity implications

The first culturally based PC consult program in the United States was developed by using CBPR, in partnership with AA and White southern rural community members. This three-phase method can serve as a model that can be replicated and adapted to other settings and with other ethnic groups; replication studies of Phase 1 are currently underway in Ghana and in Puerto Rico to determine the cultural values and preferences for care of loved ones with serious illness, of people living in those countries and cultures, another first step toward designing culturally based PC interventions by using the model developed in this study.

## Abbreviations Used

AA: African American
BMH: Beaufort Memorial Hospital
CAG: Community Advisory Group
CBPR: Community-Based Participatory Research
ICU: intensive care unit
PC: palliative care
RCT: randomized clinical trial
W: White

## References

[1] Kagawa-Singer M, Blackhall LJ Negotiating cross-cultural issues at the end of life: “you got to go where he lives.” JAMA. 2001;286:2993–3001 11743841 10.1001/jama.286.23.2993

[2] Searight HR, Gafford J Cultural diversity at the end of life: issues and guidelines for family physicians. Am Fam Physician. 2005;71:515–522 15712625

[3] Ersek M, Kagawa-Singer M, Barnes D, et al. Multicultural considerations in the use of advance directives. Oncol Nurs Forum. 1998;25:1683–1690 9826836

[4] Krakauer EL, Crenner C, Fox K Barriers to optimum end-of-life care for minority patients. J Am Geriatr Soc. 2002;50:182–190 12028266 10.1046/j.1532-5415.2002.50027.x

[5] U.S. Census Bureau. 2010 Census demographic profiles. 2010. Available at www.census.gov/2010census/popmap Accessed 817, 2017

[6] Periyakoil VS, Neri E, Kraemer H Patient-reported barriers to high-quality, end-of-life care: a multiethnic, multilingual, mixed-methods study. J Palliat Med. 2016;19:373–379 26575114 10.1089/jpm.2015.0403 PMC4827282

[7] Mazanec PM, Daly BJ, Townsend A Hospice utilization and end-of-life care decision making of African Americans. Am J Hosp Palliat Care. 2010;27:560–566 21071435 10.1177/1049909110372087

[8] Lynch S Hospice and palliative care access issues in rural areas. Am J Hosp Palliat Care. 2013;30:172–177 22531150 10.1177/1049909112444592

[9] Kwak J, Haley WE Current research findings on end-of-life decision making among racially or ethnically diverse groups. Gerontologist. 2005;45:634–641 16199398 10.1093/geront/45.5.634

[10] Mitchell BL, Mitchell LC Review of the literature on cultural competence and end-of-life treatment decisions: the role of the hospitalist. J Natl Med Assoc. 2009;101:920–926 19806850 10.1016/s0027-9684(15)31040-3

[11] Morrison RS, Augustin R, Souvanna P, et al. America's care of serious illness: a state-by-state report card on access to palliative care in our nation's hospitals. J Palliat Med. 2011;14:1094–1096 21923412 10.1089/jpm.2011.9634 PMC3391707

[12] Morrison RS, Meier DE, Rogers M, et al. America's care of serious illness: a state-by-state report card on access to palliative care in our nation's hospitals. Center to Advance Palliative Care and the National Palliative Care Research Center: New York, 2019 10.1089/jpm.2011.9634 PMC3391707 21923412

[13] Bennett KJ, Olatosi B, Probst JC Health Disparities:A Rural-Urban Chartbook. Columbia, SC: Rural Health Research Center, 2008

[14] Goodlin S Palliative care in congestive heart failure. J Am Coll Cardiol. 2009;54:386–396 19628112 10.1016/j.jacc.2009.02.078

[15] Buck HG, Riegel B The impact of frailty on health related quality of life in heart failure. Eur J Cardiovasc Nurs. 2011;10:159–166 20587372 10.1016/j.ejcnurse.2010.06.001

[16] U.S. Census Bureau. 2010 census urban and rural classification and urban area criteria. Percent rural and urban in 2010 by state [Microsoft Excel spreadsheet]. Updated November 26, 2018. Available at www.census.gov/programs-surveys/geography/guidance/geo-areas/urban-rural/2010-urban-rural.html Accessed 929, 2019

[17] U.S. Department of Agriculture Economic Research Service. State fact sheets: South Carolina. Published 2017. Updated September 6, 2019. Available at https://data.ers.usda.gov/reports.aspx?StateFIPS=45&StateName=South%20Carolina&ID=17854 Accessed 929, 2019

[18] Housing Assistance Council. Race and ethnicity in Rural America. Rural research briefs. Published 2012. Available at www.ruralhome.org/sct-information/mn-hac-research/rural-rrb/484-rrn-race-and-ethnicity Accessed 929, 2019

[19] Bakitas M, Clifford K, Dionne-Odom J, et al. Rural palliative care. In: *Oxford Textbook of Palliative Nursing, Fourth Edition*. Edited by Ferrell BR, Coyle N, Paice J. Oxford: Oxford University Press, 2015, pp. 812–822

[20] Clinical Practice Guidelines for Quality Pallative Care, 3rd ed. National Consensus Project for Quality Palliative Care: Richmond, VA, 2013

[21] Bakitas MA, Elk R, Astin M, et al. Systematic review of palliative care in the rural setting. Cancer Control. 2015;22:450–464 26678972 10.1177/107327481502200411

[22] Robinson CA, Pesut B, Bottorff JL, et al. Rural palliative care: a comprehensive review. J Palliat Med. 2009;12:253–258 10.1089/jpm.2008.0228 19216703

[23] Puri S Unequal lives, unequal deaths. In The End: The New York Times, 2016 Available at https://opinionator.blogs.nytimes.com/2016/01/20/dying-at-home-when-youre-poor/ Accessed 312, 2020

[24] Torke AM, Garas NS, Sexson W, et al. Medical care at the end of life: views of African American patients in an urban hospital. J Palliat Med. 2005;8:593–602 15992201 10.1089/jpm.2005.8.593

[25] Boucher NA, Raghavan M, Smith A, et al. Palliative care in the African American community. J Palliat Med. 2016;19:228–230 26840858 10.1089/jpm.2015.0523

[26] Check DK, Samuel CA, Rosenstein DL, et al. Investigation of racial disparities in early supportive medication use and end-of-life care among medicare beneficiaries with stage IV breast cancer. J Clin Oncol. 2016;34:2265–2270 27161968 10.1200/JCO.2015.64.8162 PMC4962709

[27] Elk R The frst step is recognizing, acknowledging, and respecting the inequity, disrespect, and disregard our African American patients have experienced. J Palliat Med. 2016;19:124–125 26840843 10.1089/jpm.2015.0524

[28] Abunafeesa H, Elsayem AF Cultural diversity and barriers to high-quality end of life care. Ann Palliat Med. 2017;6:183–186 28061533 10.21037/apm.2016.11.01

[29] Cohen LL Racial/ethnic disparities in hospice care: a systematic review. J Palliat Med. 2008;11:763–768 18588409 10.1089/jpm.2007.0216

[30] LoPresti MA, Dement F, Gold HT End-of-Life care for people with cancer from ethnic minority groups: a systematic review. Am J Hosp Palliat Care. 2014;33:291–305 25550406 10.1177/1049909114565658

[31] Payne R Racially associated disparities in hospice and palliative care access: acknowledging the facts while addressing the opportunities to improve. J Palliat Med. 2016;19:131–133 26840847 10.1089/jpm.2015.0475

[32] Rhodes RL, Teno JM, Welch LC Access to hospice for African Americans: are they informed about the option of hospice? J Palliat Med. 2006;9:268–272 16629555 10.1089/jpm.2006.9.268

[33] Colon M, Lyke J Comparison of hospice use and demographics among European Americans, African Americans, and Latinos. Am J Hosp Palliat Care. 2003;20:182–190 12785039 10.1177/104990910302000306

[34] Ludke RL, Smucker DR Racial differences in the willingness to use hospice services. J Palliat Med. 2007;10:1329–1337 18095812 10.1089/jpm.2007.0077

[35] Johnson KS, Kuchibhatla M, Tulsky JA Racial differences in self-reported exposure to information about hospice care. J Palliat Med. 2009;12:921–927 19807237 10.1089/jpm.2009.0066 PMC2904186

[36] Wicher CP, Meeker MA What influences African American end-of-life preferences? J Health Care Poor Underserved. 2012;23:28–58 22643461 10.1353/hpu.2012.0027

[37] Hazin R, Giles CA Is there a color line in death? An examination of end-of-life care in the African American community. J Natl Med Assoc. 2011;103:609–613 21999036 10.1016/s0027-9684(15)30387-4

[38] Baker ME Cultural differences in the use of advance directives: a review of the literature. Afr Am Res Perspect. 2000;6:35–40

[39] Johnson KS, Elbert-Avila KI, Tulsky JA The influence of spiritual beliefs and practices on the treatment preferences of African Americans: a review of the literature. J Am Geriatr Soc. 2005;53:711–719 15817022 10.1111/j.1532-5415.2005.53224.x

[40] True G, Phipps EJ, Braitman LE, et al. Treatment preferences and advance care planning at end of life: the role of ethnicity and spiritual coping in cancer patients. Ann Behav Med. 2005;30:174–179 16173914 10.1207/s15324796abm3002_10

[41] Ernecoff NC, Curlin FA, Buddadhumaruk P, et al. Health care professionals' responses to religious or spiritual statements by surrogate decision makers during goals-of-care discussions. JAMA Intern Med. 2015;175:1662–1669 26322823 10.1001/jamainternmed.2015.4124

[42] Kennard CL Undying hope. J Palliat Med. 2016;19:129–130 26840846 10.1089/jpm.2015.0331

[43] Mansfield CJ, Mitchell J, King DE The doctor as God's mechanic? Beliefs in the Southeastern United States. Soc Sci Med. 2002;54:399–409 11824916 10.1016/s0277-9536(01)00038-7

[44] Bailey FA, Burgio KL, Woodby LL, et al. Improving processes of hospital care during the last hours of life. Arch Intern Med. 2005;165:1722–1727 16087819 10.1001/archinte.165.15.1722

[45] Abrahm JL, Callahan J, Rossetti K, et al. The impact of a hospice consultation team on the care of veterans with advanced cancer. J Pain Symptom Manage. 1996;12:23–31 8718913 10.1016/0885-3924(96)00045-0

[46] Bascom PB A hospital-based comfort care team: consultation for seriously ill and dying patients. Am J Hosp Palliat Care. 1997;14:57–60 9295403 10.1177/104990919701400202

[47] Kuin A, Courtens AM, Deliens L, et al. Palliative care consultation in The Netherlands: a nationwide evaluation study. J Pain Symptom Manage. 2004;27:53–60 14711469 10.1016/j.jpainsymman.2003.06.001

[48] Manfredi PL, Morrison RS, Morris J, et al. Palliative care consultations: how do they impact the care of hospitalized patients? J Pain Symptom Manage. 2000;20:166–173 11018334 10.1016/s0885-3924(00)00163-9

[49] Bakitas M, Stevens M, Ahles T, et al. Project ENABLE: a palliative care demonstration project for advanced cancer patients in three settings. J Palliat Med. 2004;7:363–372 15130218 10.1089/109662104773709530

[50] Bakitas M, Lyons KD, Hegel MT, et al. The project ENABLE II randomized controlled trial to improve palliative care for rural patients with advanced cancer: baseline findings, methodological challenges, and solutions. Palliat Support Care. 2009;7:75–86 19619377 10.1017/S1478951509000108 PMC3685415

[51] Elsayem A, Smith ML, Parmley L, et al. Impact of a palliative care service on in-hospital mortality in a comprehensive cancer center. J Palliat Med. 2006;9:894–902 16910804 10.1089/jpm.2006.9.894

[52] Elliott AM, Alexander SC, Mescher CA, et al. Differences in physicians' verbal and nonverbal communication with black and white patients at the end of life. J Pain Symptom Manage. 2016;51:1–8 26297851 10.1016/j.jpainsymman.2015.07.008 PMC4698224

[53] Halloway K Their bodies, our conduct: how society and medicine produce persons standing in need of end of life care. J Palliat Med. 2016;19:127–128 26840845 10.1089/jpm.2015.0256 PMC4753580

[54] Sue D Microaggressions in Everyday Life: Race, Gender and Sexual Orientation. Hoboken, NJ: John Wiley and Sons, 2010

[55] Freeman HP, Payne R Racial injustice in health care. N Engl J Med. 2000;342:1045–1047 10749970 10.1056/NEJM200004063421411

[56] Shrank WH, Kutner JS, Richardson T, et al. Focus group findings about the influence of culture on communication preferences in end-of-life care. J Gen Intern Med. 2005;20:703–709 16050878 10.1111/j.1525-1497.2005.0151.x PMC1490193

[57] Taxis JC Attitudes, values, and questions of African Americans regarding participation in hospice programs. J Hosp Palliat Nurs. 2006;2:77–85

[58] Goldberg L Poll: doctors want to discuss end-of-life issues, but barriers remain. In: Research & Analysis. The Pew Charitable Trusts, 2016 Available at www.pewtrusts.org Accessed 126, 2019

[59] Webster Dictionary.org “Deep South.” Available at www.webster-dictionary.org/definition/Deep%20South Accessed 817, 2016

[60] Sewell AA Disaggregating ethnoracial disparities in physician trust. Soc Sci Res. 2015;54:1–20 26463531 10.1016/j.ssresearch.2015.06.020

[61] U.S. Census Bureau. State and county quickfacts (Beaufort County, South Carolina). Published 2015 Available at http://quickfacts.census.gov/qfd/states/45/45013.html Accessed 728, 2015

[62] Chau TS, Islam N, Tandon D, et al. Using community-based participatory research as a guiding framework for health disparities research centers. Prog Community Health Partnersh. 2007;1:195–205 19081761 10.1353/cpr.2007.0007 PMC2600476

[63] Wallerstein NB, Duran B Using community-based participatory research to address health disparities. Health Promot Pract. 2006;7:312–323 16760238 10.1177/1524839906289376

[64] Laveaux D, Christopher S Contextualizing CBPR: key principles of CBPR meet the indigenous research context. Pimatisiwin. 2009;7:1 20150951 PMC2818123

[65] Israel BA, Eng E, Schulz AJ, et al. Methods in Community-Based Participatory Research for Health, 1st ed. San Francisco, CA: Jossey-Bass, 2005

[66] Riffin C, Kenien C, Ghesquiere A, et al. Community-based participatory research: understanding a promising approach to addressing knowledge gaps in palliative care. Ann Palliat Med. 2016;5:218–224 27481321 10.21037/apm.2016.05.03 PMC5470584

[67] Halcomb EJ, Gholizadeh L, DiGiacomo et al. Literature review: considerations in undertaking focus group research with culturally and linguistically diverse groups. J Clin Nurs. 2007;16:1000–1011 17518876 10.1111/j.1365-2702.2006.01760.x

[68] Krueger RA Focus Groups: A Practical Guide for Applied Research, 2nd ed. Thousands Oaks, CA: Sage Publications, 1994

[69] Fox K, Hinton WL, Levkoff S Take up the caregiver's burden: stories of care for urban African American elders with dementia. Cult Med Psychiatry. 1999;23:501–529 10647946 10.1023/a:1005520105518

[70] Martin A Health Systems Profile for Beaufort, Jasper, and Hampton Counties. Columbia, SC: Arnold School of Public Health, University of South Carolina, 2011

[71] Leite R, Hudson C, West L, et al. Assessment of oral health needs and barriers to care in a Gullah community: hollywood smiles. Prog Community Health Partnersh. 2013;7:201–208 23793251 10.1353/cpr.2013.0016 PMC4097834

[72] Hinton L, Franz CE, Yeo G, et al. Conceptions of dementia in a multiethnic sample of family caregivers. J Am Geriatr Soc. 2005;53:1405–1410 16078970 10.1111/j.1532-5415.2005.53409.x

[73] Merriam SB Qualitative Research: A Guide to Design and Implementation. San Francisco, CA: Jossey-Bass, 2009

[74] Donabedian A The quality of care: how can it be assessed? JAMA. 1988;260:1743–1748 3045356 10.1001/jama.260.12.1743

[75] Johnson CL, Barer BM Families and networks among older inner-city blacks. Gerontologist. 1990;30:726–733 2286330 10.1093/geront/30.6.726

[76] Taylor RJ, Chatters LM, Woodward AT, et al. Racial and ethnic differences in extended family, friendship, fictive kin and congregational informal support networks. Fam Relat. 2013;62:609–624 25089067 10.1111/fare.12030 PMC4116141

[77] Chatters LM, Taylor RJ, Jayakody R Fictive kinship relations in black extended families. J Comp Fam Stud. 1994;25:297–312

[78] Thrall JH, Boland G Telemedicine in practice. Semin Nucl Med. 1998;28:145–157 9579416 10.1016/s0001-2998(98)80004-4

[79] Bjorn P Rural teletrauma: applications, opportunities, and challenges. Adv Emerg Nurs J. 2012;34:232–237 22842966 10.1097/TME.0b013e31825f6237

[80] Hess DC, Wang S, Hamilton W, et al. REACH: clinical feasibility of a rural telestroke network. Stroke. 2005;36:2018–2020 16051892 10.1161/01.STR.0000177534.02969.e4

[81] Steventon A, Bardsley M, Billings J, et al. Effect of telehealth on use of secondary care and mortality: findings from the Whole System Demonstrator cluster randomised trial. BMJ. 2012;344:e3874 22723612 10.1136/bmj.e3874 PMC3381047

[82] Stradling DA Telestroke: state of the science and steps for implementation. Crit Care Nurs Clin North Am. 2009;21:541–548 19951769 10.1016/j.ccell.2009.07.017

[83] Taylor DM, Stone SD, Huijbregts MP Remote participants' experiences with a group-based stroke self-management program using videoconference technology. Rural Remote Health. 2012;12:1947 22463728

[84] National Quality Forum. Creating a Framework to Support Measure Development for Telehealth. National Quality Forum: Washington, DC, 2017

[85] Aoun S, Bird S, Kristjanson LJ, et al. Reliability testing of the FAMCARE-2 scale: measuring family carer satisfaction with palliative care. J Palliat Med. 2010;24:674–681 10.1177/0269216310373166 20621947

[86] Whitehead K Lived religion in the south: emerging narratives. Americanist. 2012;27:16

[87] Harvey P Race, culture, and religion in the American South. Oxford Research Encyclopedias. 2015 Available at https://oxfordre.com/religion/view/10.1093/acrefore/9780199340378.001.0001/acrefore-9780199340378-e-7?rskey=JwymNs&result=28 Accessed 312, 2020

[88] Reese DJ, Ahern RE, Nair S, et al. Hospice access and use by African Americans: addressing cultural and institutional barriers through participatory action research. Soc Work. 1999;44:549–559 10568027 10.1093/sw/44.6.549

[89] Pipes W. Old-time religion: benches can't say “Amen.” In: Blackfamilies. Edited by McAdoo H. Beverly Hills, CA: Sage Publications, 1981, pp. 54–76

[90] Johnson J, Hayden T, True J, et al. The impact of faith beliefs on perceptions of end-of-life care and decision making among African American church members. J Palliat Med. 2016;19:143–148 26840849 10.1089/jpm.2015.0238

[91] Dilworth-Anderson P, Williams IC, Gibson BE Issues of race, ethnicity, and culture in caregiving research: a 20-year review (1980–2000). Gerontologist. 2002;42:237–272 11914467 10.1093/geront/42.2.237

[92] Brummett BH, Siegler IC, Williams RB, et al. Associations of social support and 8-year follow-up depressive symptoms: differences in African American and White caregivers. Clin Gerontol. 2012;35:289–302 23144529 10.1080/07317115.2012.678569 PMC3491574

[93] Dilworth-Anderson P, Brummett BH, Goodwin P, et al. Effect of race on cultural justifications for caregiving. Gerontology Ser B. 2005;60:S257–S262 10.1093/geronb/60.5.s257 16131626

[94] Dilworth-Anderson P, Pierre G, Hilliard TS Social justice, health disparities, and culture in the care of the elderly. J Law Med Ethics. 2012;40:26–32 22458459 10.1111/j.1748-720X.2012.00642.x

[95] Burton LM, Dilworth-Anderson P The intergenerational family roles of aged black Americans. Marriage Fam Rev. 2008;16:311–330

[96] Elk R, Landrine H Cancer Disparities: Causes and Evidence-Based Solutions. New York, NY: Springer Publishing Co., 2011

